# Smoking as a risk factor for rheumatoid arthritis: predominant association with IgA autoantibodies – comprehensive analysis of anti-modified protein antibodies with smoking and genetic risk factors in rheumatoid arthritis

**DOI:** 10.1186/s13075-025-03543-6

**Published:** 2025-05-08

**Authors:** Tineke J. van Wesemael, Fraser R. Morton, Judith W. Heutz, Marc P. Maurits, Annemarie L. Dorjée, Duncan Porter, Tom W. J. Huizinga, Karim Raza, Rene E. M. Toes, Rachel Knevel, Pascal H. P. de Jong, Anna Svärd, Diane van der Woude

**Affiliations:** 1 Department of Rheumatology, Leiden University Medical Center, Albinusdreef 2, Leiden, 233ZA the Netherlands; 2 School of Infection and Immunity, University of Glasgow, University Avenue, Glasgow, G12 1QQ UK; 3 Department of Rheumatology, Erasmus Medical Center, Rotterdam, The Netherlands; 4 Rheumatology Research Group, Institute of Inflammation and Ageing, College of Medical and Dental Sciences, University of Birmingham, Birmingham, UK; 5 Department of Rheumatology, Bronglais Hospital, Hywel Dda University Health Board, Aberystwyth, UK; 6 National Institute for Health and Care Research (NIHR) Birmingham Biomedical Research Centre, Birmingham, UK; 7 Center for Clinical Research Dalarna, Uppsala University, Falun, Sweden

**Keywords:** Rheumatoid arthritis, Anti-modified protein antibodies, Anti-acetylated protein antibodies, Anti-citrullinated protein antibodies, Smoking, Genetic risk factors

## Abstract

**Background:**

Rheumatoid arthritis (RA) is an autoimmune disease characterized by the presence of autoantibodies against modified proteins, known as anti-modified protein autoantibodies (AMPAs). While the relationship between different AMPA isotypes and various risk factors remains poorly understood, investigating this association is important for a deeper understanding of RA pathophysiology. Smoking, has its primary effects in the lungs, and it remains unclear whether smoking is preferentially linked to specific AMPA isotypes, such as IgA, which could suggest a mucosal origin. Therefore, we set out to investigate the association between smoking, genetic risk factors for RA, and the presence of specific AMPA isotypes, particular IgA.

**Methods:**

In 618 RA patients, anti-citrullinated protein antibodies (ACPA-) and anti-acetylated protein antibodies (AAPA-) IgA, -IgG and -IgM and RF-IgA and -IgM were measured by ELISA. Associations with genetic risk factors, smoking and autoantibodies were assessed with logistic regression analysis. For replication, a comprehensive meta-analysis incorporating 3309 RA patients was performed.

**Results:**

Smoking was primarily associated with IgA AMPA, with associations that prevailed after correcting for the concurrent presence of AMPA IgG (ACPA-IgA OR 1.89 [1.14–3.12], AAPA-IgA 2.30 [1.35–3.94]). To further substantiate these results, we performed a meta-analysis of 3309 RA patients and observed that smoking was again predominantly associated with the combined presence of ACPA-IgA in addition to ACPA-IgG (OR 2.05 [1.69–2.49], *p* < 0.001) versus the single presence of ACPA-IgG (OR 1.18 [0.97–1.44], *p* = 0.11). A gene-environment interaction between the most important genetic risk factor for RA (the HLA shared epitope alleles) and smoking was only seen in patients that were both ACPA-IgG and ACPA-IgA positive, but not in patients who were only positive for ACPA-IgG.

**Conclusion:**

These data provide a pivotal refinement of existing knowledge regarding risk factor associations for RA and lend novel support to the hypothesis that smoking may exert its effect on RA by the induction of local (auto)immune responses at mucosal sites.

**Supplementary Information:**

The online version contains supplementary material available at 10.1186/s13075-025-03543-6.

## Background

Rheumatoid arthritis (RA) is characterized by the presence of autoantibodies such as rheumatoid factor (RF) and antibodies against post translationally modified proteins (also known as anti-modified protein antibodies (AMPAs) [1]. AMPAs found in RA patients are anti-citrullinated protein antibodies (ACPA), anti-carbamylated protein antibodies (anti-CarP) and anti-acetylated protein antibodies (AAPA) [2]. Patients with and without autoantibodies differ with regard to risk factors for developing disease: the vast majority of genetic risk factors predispose to ACPA-positive RA, such as the HLA-DRB1 shared epitope alleles (SE) and Protein tyrosine phosphatase non-receptor type 22 (PTPN22)-variants [3, 4]. Smoking is the best studied environmental risk factor and is associated with RF and the concurrent presence of multiple AMPA-IgG [5, 6]. A gene-environment interaction between SE and smoking has been found in ACPA-IgG positive patients providing supportive evidence for the interesting hypothesis that autoimmunity in RA has a mucosal origin [4, 7].

The mucosal origin hypothesis postulates that the first trigger in RA development is at the mucosal sites of for instance the lung, oral cavity or gut where antibodies are produced locally. These antibodies could subsequently cross-react with self-antigens in joints leading to disease onset. In light of the gene-environment interaction described above, this hypothesis can be specified for the lungs in the sense that smoking can lead to increased citrullination in pulmonary tissue possibly followed by presentation of these citrullinated antigens to HLA‐SE‐restricted T cells, which could lead to local T and B cell activation. This hypothesis is supported by the following findings: (i) smokers appear to have more citrullinated proteins in broncho-alveolar lavage (BAL) fluid than non-smokers [8] and (ii) ACPA-positive B cells and ACPA have been found in the sputum of RA patients and persons at risk of developing RA, suggesting local production of ACPA [9, 10]. Since mucosal immune responses are characterized by production of IgA it would be of great interest to study the association of AMPA-IgA with risk factors for RA. Most studies on risk factors thus far have focused on ACPA-IgG, while the association of risk factors with ACPA-IgA remains largely uninvestigated and thus poorly understood.

In the context of the mucosal origin hypothesis, the new anti-acetylated protein antibodies (AAPA) are particularly interesting. It appears plausible that acetylated antigens could be abundantly present at mucosal surfaces since many bacteria possess enzymes capable of acetylating self- and host proteins [11]. It would be of great interest to study the association of AAPA isotypes with known risk factors such as genetic predisposition and smoking, since it would increase our understanding of pathological mechanisms underlying the development of this autoantibody and its isotypes. This is of special interest since AMPA are to a certain extent cross-reactive, although there are patients and autoantibody clones that specifically recognize one of these post-translational modifications [12–15]. Uncovering unique versus shared associations between AMPA will shed light on the unique and shared etiological mechanisms underlying the development of the different AMPAs.

Therefore, the aim of the study was to explore the association between smoking, genetic risk factors and the presence of IgA and/or IgG AMPA in RA patients, to determine whether different risk factors and underlying mechanisms predispose to the development of AMPA of a specific reactivity and isotype.

## Methods

### Population

In three different cohorts, the Leiden early arthritis clinic (EAC) and the “treatment in the Rotterdam Early Arthritis Cohort” (tREACH) from the Netherlands and the Scottish Early Rheumatoid Arthritis (SERA)-inception cohort from Scotland patients with RA were investigated [16–20]. In the EAC, all patients fulfilled the 1987 ACR/EULAR RA classification criteria, in the tREACH either the 1987 or 2010 ACR/EULAR RA classification criteria and in the SERA the 2010 ACR/EULAR RA classification criteria. Cohorts are further described in the supplementary materials.

The protocol of each cohort was approved by the relevant local ethics committee and all participants provided written informed consent.

### Detection of antibodies

All antibodies in the EAC, tREACH and SERA were measured in baseline serum samples (collected at disease onset) [17, 21, 22]. The cut-off for autoantibody detection was determined based on the manufacturer's guidelines or, for in-house ELISAs, established to ensure comparable specificity across all autoantibodies. A maximum of 2.5% of healthy controls tested positive, corresponding to a specificity of 97.5%. Detailed methodologies for autoantibody measurements are provided in the supplementary file.

### Clinical data

In the EAC, information about demographics, symptom duration, family history and disease characteristics was recorded at baseline [16]. Data concerning smoking were collected at baseline using a questionnaire. In the entire cohort, smoking was defined as the current smoking of cigarettes versus past and never smoking, since the distinction between never smoking and past smoking was not recorded in patients included before 2003. No data on pack-years were available.

In the tREACH data on smoking were collected at baseline using a questionnaire. Patients could be categorized as smokers, past smokers and non-smokers.

In SERA, patients were categorized as smokers, past smokers and non-smokers.

### Genetic risk factors

In the EAC, HLA-DRB1 genotyping was performed as described previously in 648 RA patients and 1211 controls [23]. The SERA participants and the controls were genotyped by deCODE genetics using Illumina’s GSA v3 array with deCODE’s customized drop-in. The arrays were processed using PLINK and HLA imputation was performed using CookHLA [24]. HLA-DRB1 * 01:01, HLA-DRB1 * 01:02, HLA-DRB1 * 04:01, HLA-DRB1 * 04:04, HLA-DRB1 * 04:05, HLA-DRB1 * 04:08 and/or HLA-DRB1 * 10:01, were classified as the SE alleles. In the tREACH genetic data were not available.

In the EAC, information on the PTPN22-SNP rs2476601 and a polygenetic risk score (PRS) were available. PTPN22 SNP-typing was available in 361 RA patients and 411 controls, as described elsewhere [25]. Genotyping was performed on either the Illumina Global Screening Array or Illumina ImmunoChip and the PRS included 85 SNPs as described before [26]. The PRS was available in 543 RA patients. The number of patients with available data for HLA-DR-B1 alleles, PTPN22, and PRS varied, with no indication of any systematic bias.

### Sample size

As this is a cohort study with a fixed number of patients, we conducted a power calculation to determine the feasibility of subgroup analyses. For instance, to detect an association between smoking and autoantibodies in the EAC, with an alpha of 0.05, a power of 0.80 and expected odds ratio (OR) for the association of smoking with autoantibody positivity of 1.5 (based on earlier findings) [6] we were able to split the groups for two autoantibodies, but not for more than two due to the power limitations associated with this sample size.

### Literature search for meta-analysis

For the meta-analyses of the association between smoking and ACPA-isotypes, and the association between SE and AAPA-IgG a systematic literature review was performed in PubMed, as described in the supplementary material, Figure S1 and Figure S2.

### Statistical analysis

Antibody levels among different subgroups were compared using Mann–Whitney U tests. The association between autoantibodies and smoking, genetic risk factors and phenotype at baseline was investigated using logistic regression analysis. For genetic outcomes, healthy individuals were used as the reference group. For all other analyses the autoantibody negative patients who did not have the risk factor under study were the reference group. The presence of biological interaction between smoking and SE alleles, was defined as the deviation from additivity. This was assessed by the attributable proportion due to interaction (AP), relative excess risk due to interaction (RERI) and Synergy index (S), and was calculated as described previously [27, 28]. For the meta-analysis of the association between smoking with antibodies a fixed-effects model was used since smoking prevalence was more or less comparable between the cohorts and there was no significant statistical heterogeneity as assessed by the Q-statistic. For the meta-analysis of the association between SE with antibodies a random-effects model was used since the effect size of SE is expected to differ between ethnicities.

The selected patient group for this study all had data available on ACPA and AAPA isotypes, smoking, and SE, ensuring that missing data were not an issue in the majority of analyses. For some variables, such as PRS, anti-CarP IgA, and PTPN22, measurements were only available for a subset of patients, with no indications of systematic bias. Statistical analysis in the EAC were performed in STATA (version 16), in the tREACH in STATA (version 17) and in the SERA in R.

## Results

### Frequency of AMPA isotypes

**Fig. 1:**
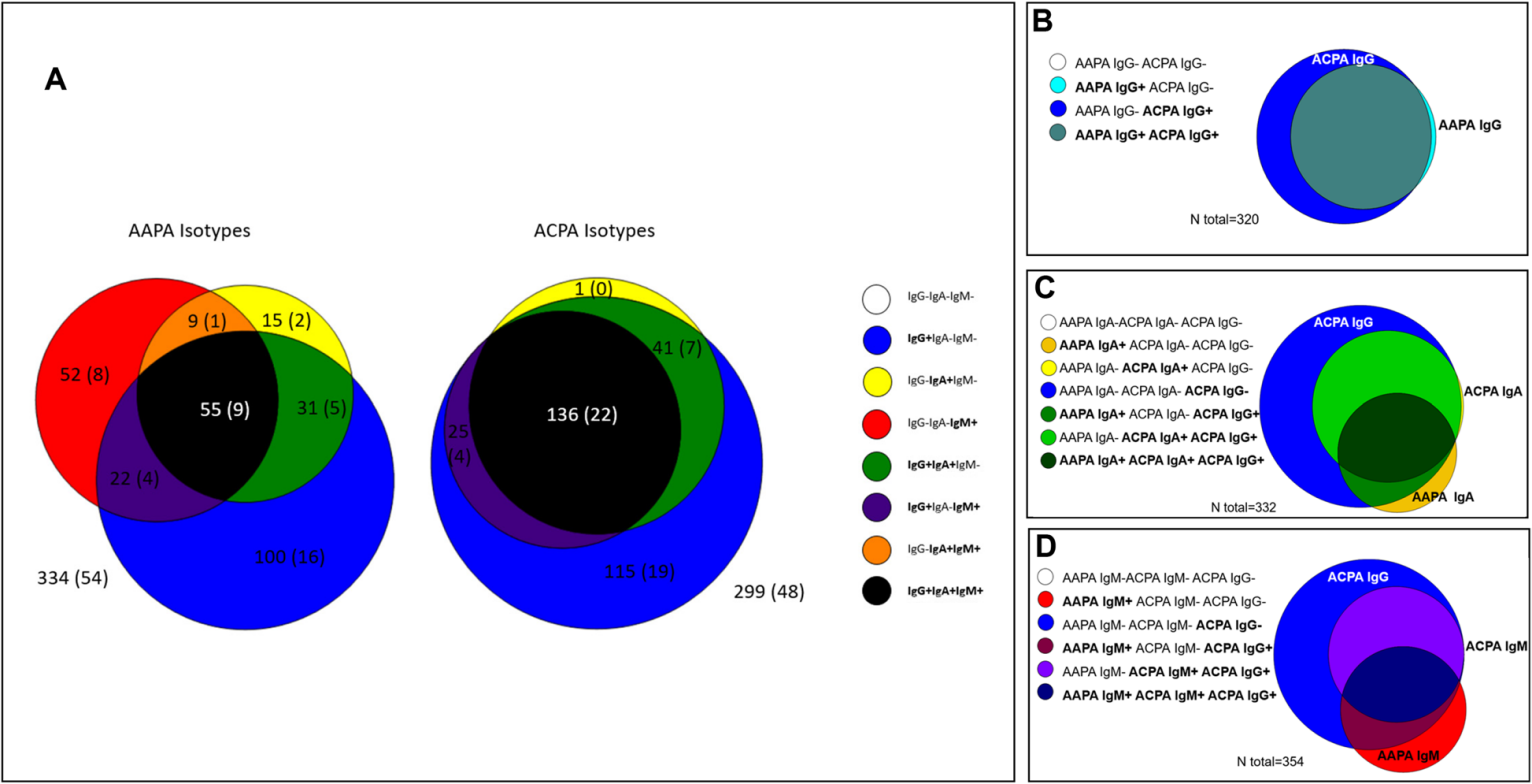
Prevalence of ACPA and AAPA isotypes in the EAC. **A** prevalence of AAPA isotypes and ACPA isotypes. **B** prevalence of AAPA & ACPA IgG. **C** prevalence of AAPA & ACPA IgA. **D** prevalence of AAPA & ACPA IgM. AAPA: anti-acetylated protein antibodies, ACPA: anti-citrullinated protein antibodies, EAC: Leiden early arthritis clinic

In this study, we investigated the association of AMPA isotypes with smoking and genetic risk factors in RA patients from the Leiden EAC, SERA, and tREACH cohorts, with primary analyses conducted in the EAC cohort and replication of findings performed using the SERA and tREACH cohorts. Baseline characteristics of the cohorts are described in Table S1. First AMPA isotype frequency was studied in the EAC: ACPA-IgG was found in 317 (51%) and AAPA-IgG in 208 patients (34%). Table S2 and Fig. 1 depict the presence and overlap of the various AMPA-isotypes in the EAC. Interestingly, for AAPA, IgM and IgA were found in IgG negative patients (8% and 2% respectively), whereas for ACPA, IgA and IgM were almost exclusively found in IgG positive patients (Fig. 1A). The number of patients that was AAPA-IgG positive, but ACPA-IgG negative was very limited: 4 (1%) (Fig. 1B). As shown in Fig. 1C and D, AAPA-IgA and AAPA-IgM were also present in patients who were ACPA-IgA and ACPA-IgM negative.

### Association of smoking with AMPA isotypes

**Fig. 2:**
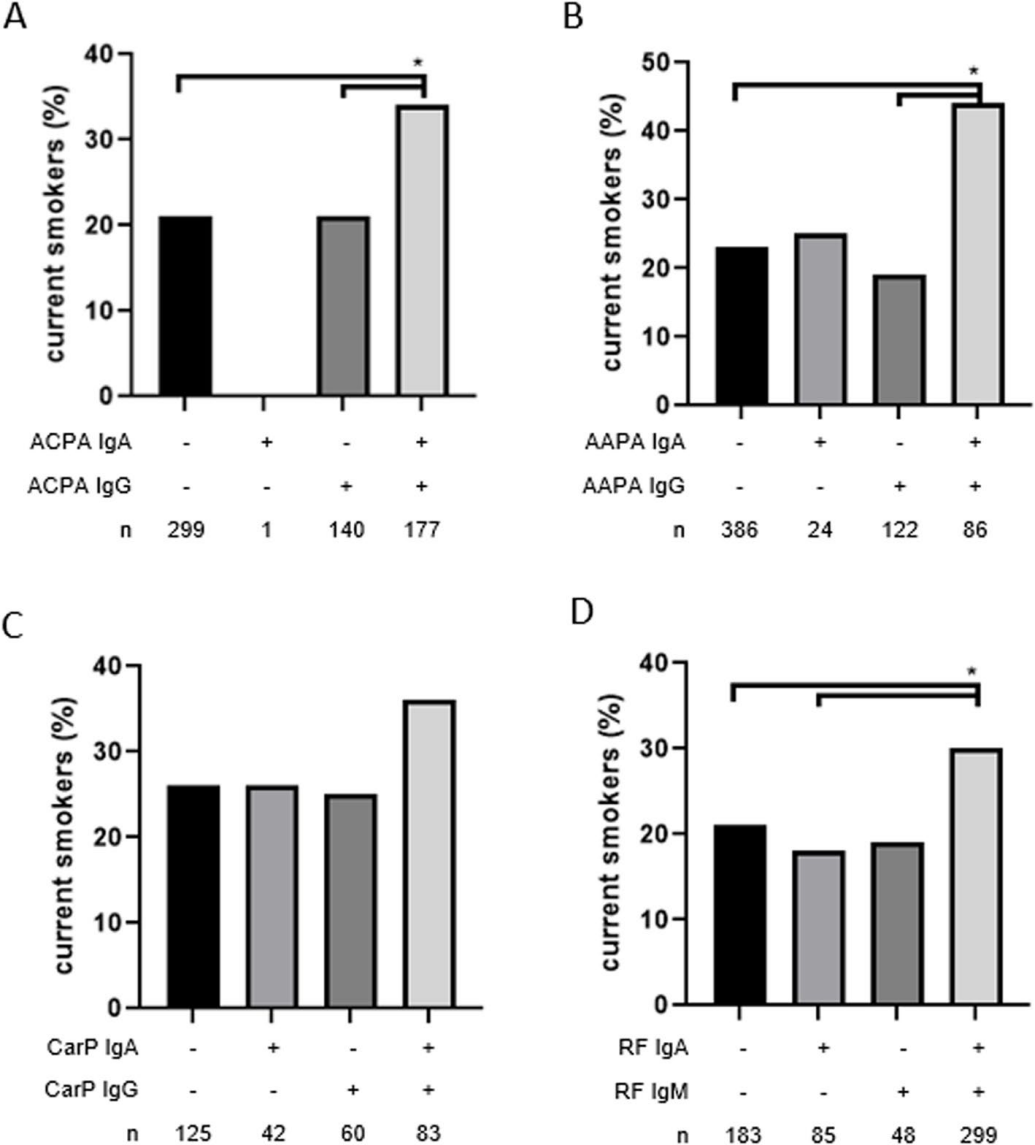
Proportion of smoking patients in relation to anti-modified protein antibodies and rheumatoid factor in the Leiden EAC. **A** Proportion of smoking with ACPA IgA & ACPA IgG. **B** Proportion of smoking with AAPA IgA & AAPA IgG. **C** Proportion of smoking with CarP IgA & CarP IgG. **D** Proportion of smoking with RF IgA & RF IgM. * *p*-value < 0.05. AAPA: anti-acetylated protein antibodies, ACPA: anti-citrullinated protein antibodies, CarP: anti-carbamylated protein antibodies, EAC: Leiden early arthritis clinic, RF: rheumatoid factor

To investigate whether smoking is associated with IgA autoantibodies, we studied the association between smoking and RF- and AMPA-isotypes, and IgA in particular in the Leiden EAC. In univariate analysis, smoking was associated with various autoantibodies of different isotypes (ACPA-IgG, ACPA-IgA, AAPA-IgA, RF-IgM and RF-IgA), with slightly stronger associations with ACPA and AAPA IgA compared to IgG (Table S3). However, since isotypes often co-occur, this precluded firm conclusions about specific associations with one autoantibody versus others. Therefore, we analyzed next the composition of autoantibody isotypes, focusing on the presence of IgA alone or in combination with other isotypes. This analysis showed that smoking was only associated with autoantibodies in patients who were double-positive, meaning those who tested positive for both IgG and IgA-AMPA or for both IgM and IgA-RF (Fig. 2, Table S4). The simultaneous presence of several autoantibody isotypes is also associated with higher levels of these autoantibodies (data not shown). To investigate whether the association with smoking could be ascribed to the presence of IgA-autoantibodies or rather to higher autoantibody levels, we next corrected the isotype-smoking associations for these levels. Strikingly, after correction for AMPA-IgA, the association between AMPA-IgG and smoking was lost, whereas smoking was still associated with ACPA-IgA and AAPA-IgA after correction for IgG suggesting a more specific association of smoking with IgA than IgG autoantibodies (Figure S3A, S3B, S3C and Table S5).

For RF, the findings were slightly different: the association of smoking with both RF-IgM and -IgA was lost when correcting for levels of the other isotype (correcting RF-IgM for RF-IgA and RF-IgA for RF-IgM) (Figure S3D and Table S5). Further analysis (detailed in the supplementary results) revealed that the association of smoking with RF was based on the presence of ACPA-IgA, indicating that the previously found association of smoking with RF-IgM and RF-IgA is caused by confounding due to ACPA-IgA (Table S6, Table S7). In summary therefore, the data indicate that smoking is associated with AMPA-IgA.

To investigate if the definition of smoking as used in the analyses above (current versus never and past) led to an underestimation of the effect of smoking, we then carried out a sub-analysis in patients in whom more detailed smoking information was available: 234 patients included in the EAC after 2003. Surprisingly, when smoking was categorized as ever versus never, there was no association with any autoantibody (Table S8). A detailed analysis of current versus past versus never smoking revealed that only current smoking was associated with autoantibody positivity in the EAC (Table S8). We strove to replicate these findings in an independent cohort and thus obtained unpublished data from the Scottish SERA and the Dutch tREACH cohort. Also in these populations, current smoking was most clearly associated with autoantibody positivity (Table S8).

**Fig. 3:**
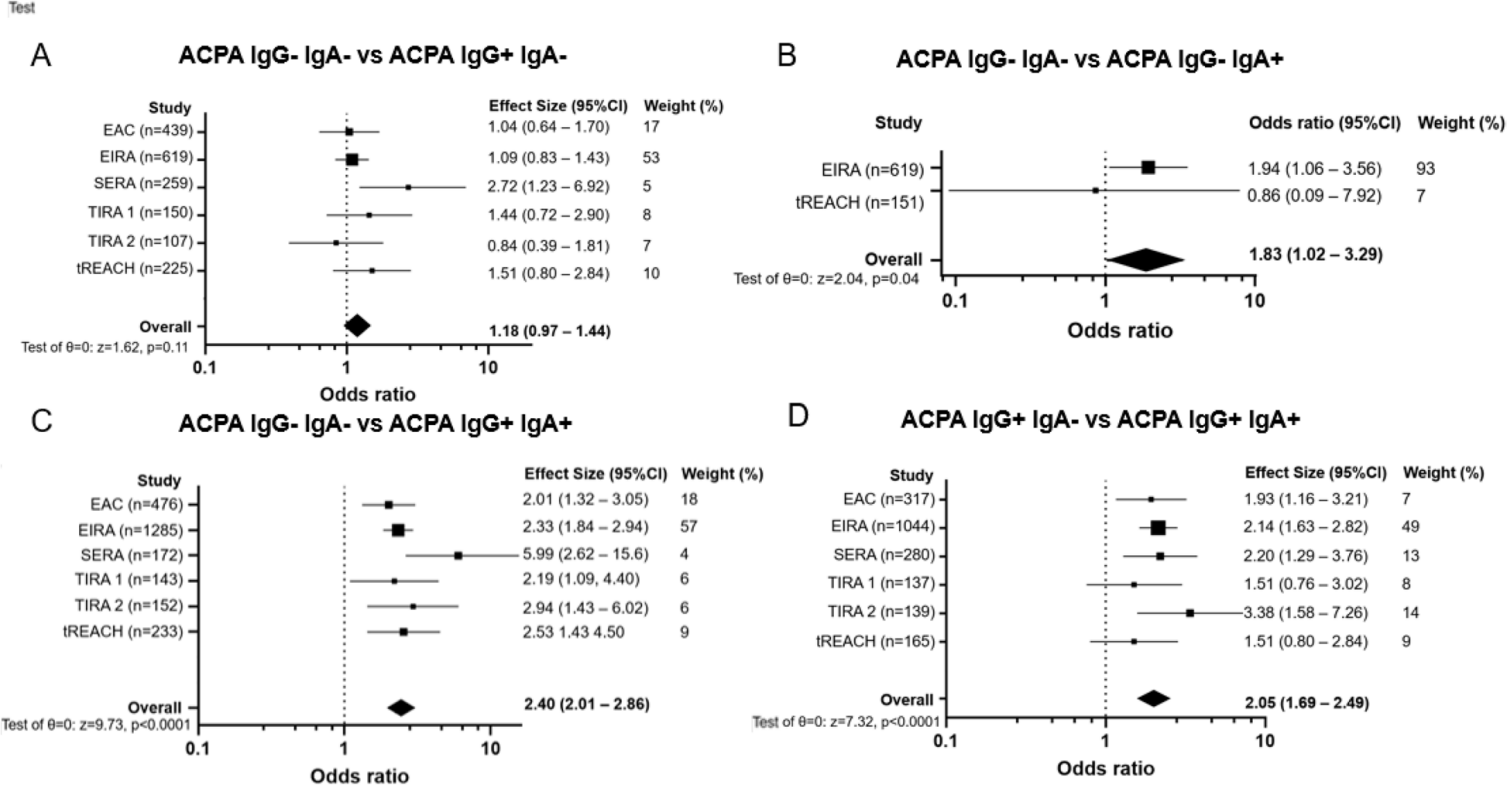
Meta-analysis combining study data with SLR for the association of smoking^*^ with ACPA-IgG and ACPA-IgA. **A** Meta-analysis for the association of smoking: ACPA-IgG- ACPA-IgA- versus ACPA-IgG + ACPA-IgA- **B**. Meta-analysis for the association of smoking: ACPA-IgG- ACPA-IgA- versus ACPA-IgG- ACPA-IgA + **C**. Meta-analysis for the association of smoking: ACPA-IgG- ACPA-IgA- versus ACPA-IgG + ACPA-IgA + **D**. Meta-analysis for the association of smoking: ACPA-IgG + ACPA-IgA- versus ACPA-IgG + ACPA-IgA +. *Smoking was defined as current versus never and past in EAC, SERA and tREACH and as ever versus never in the EIRA, TIRA- 1 and TIRA- 2. ACPA: anti-citrullinated protein antibodies. EAC: Leiden early arthritis clinic, EIRA: Epidemiological Investigations of RA [29], SERA: Scottish Early Rheumatoid Arthritis, TIRA 1: Early Intervention in RA 1, TIRA 2: Early Intervention in RA 2 [29], tREACH: treatment in the Rotterdam Early Arthritis Cohort

To confirm whether smoking is associated with ACPA-IgA, we validated our findings using additional data. We included unpublished data from the tREACH and SERA cohorts and conducted a systematic literature review on the relationship between smoking and ACPA isotype composition (Figure S1). We then performed a meta-analysis combining data from the literature with results from the EAC, SERA, and tREACH cohorts. Previous analyses showed a stronger association with smoking in current smokers. Therefore, in the EAC, SERA, and tREACH cohorts, smoking was categorized as ‘current smokers’ versus ‘past/never smokers’. In studies from the literature, smoking data was only available as ‘ever’ versus ‘never smokers’. The meta-analysis revealed no significant association between smoking and ACPA-IgG single-positive RA (odds ratio [OR] 1.18 [0.97–1.44]; Fig. 3A). However, there was a significant association with ACPA-IgA single-positive RA (OR 1.83 [1.02–3.29]; Fig. 3B). A stronger association was found for double-positive patients (ACPA-IgA + ACPA-IgG +; OR 2.40 [2.01–2.86]; Fig. 3C). To further explore the added value of ACPA-IgA, we compared ACPA-IgG single-positive RA to double-positive RA. This analysis showed a clear association between smoking and double-positive RA (OR 2.05 [1.69–2.49]; Fig. 3D), reinforcing the link between smoking and ACPA-IgA.

**Fig. 4:**
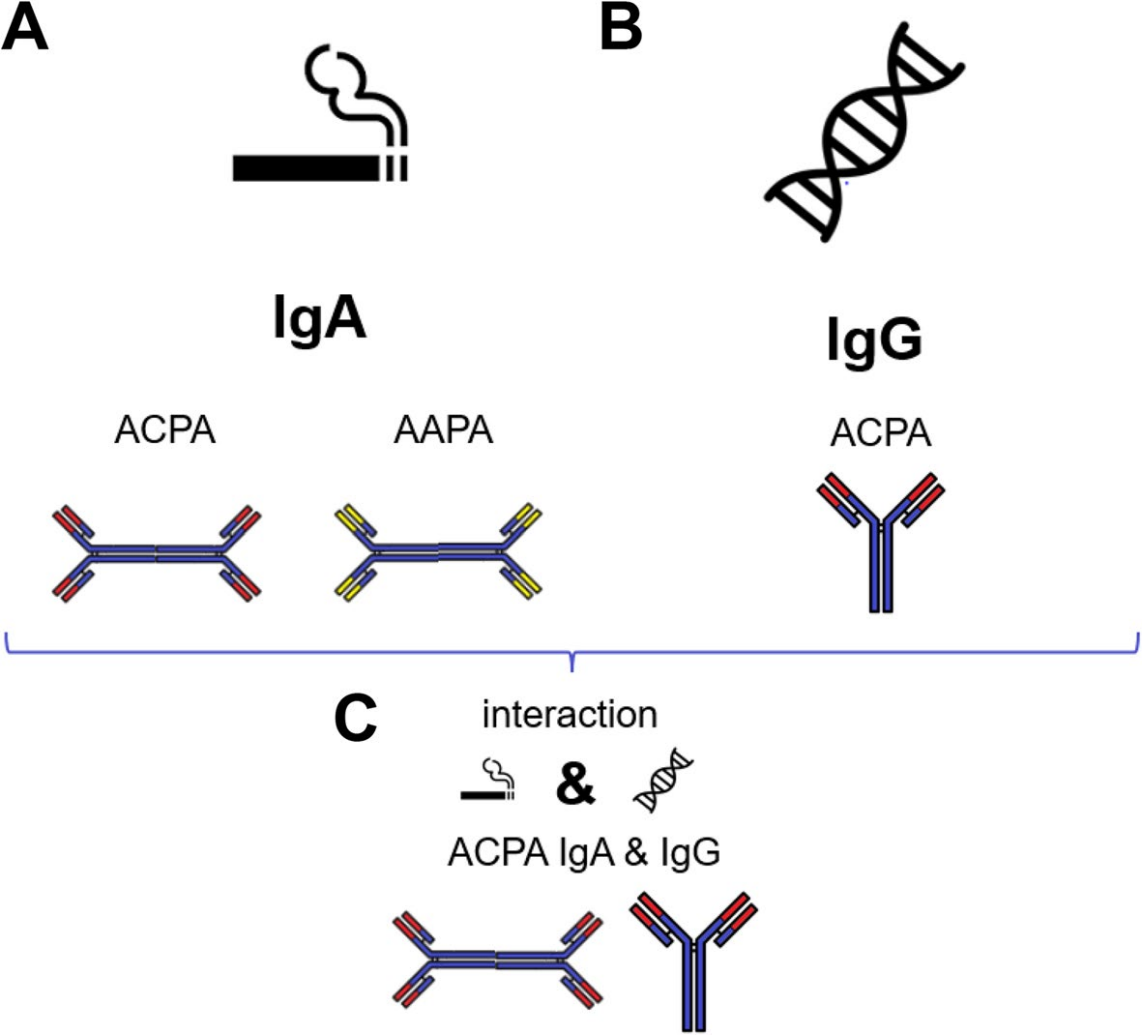
Association of smoking and shared epitope with anti-modified protein antibodies isotypes in rheumatoid arthritis. AAPA: anti-acetylated protein antibodies, ACPA: anti-citrullinated protein antibodies

Sub meta-analysis with different definitions of smoking were performed (e.g. smoking ever versus smoking never and smoking current versus smoking past), with similar results as the meta-analysis presented above (Figure S4 and Figure S5). This further reinforces that the association of smoking is mainly with ACPA-IgA, both in the presence and absence of ACPA-IgG (Fig. 4A).

### Association of AMPA isotypes with genetic risk factors

To further elucidate the associations between known risk factors for RA and specific AMPA isotypes, we then focused our attention on the most potent genetic risk factor for RA: the SE alleles. As has been described before, a strong association of SE with ACPA-IgG positivity as compared to healthy controls was found with an OR 4.75 [3.54–6.38] (Table 1). This association was significantly stronger in patients with two copies of SE compared to one copy, with an OR of 2.21 (1.19–4.13), *p* = 0.01 (Table S9). In univariate analysis, SE was also associated with AAPA-IgG (Table 1), as well as with ACPA-IgA, ACPA-IgM, AAPA-IgA and AAPA-IgM (Table S10). To investigate whether these associations were independent of the well-known association of SE with ACPA-IgG, analyses were stratified for ACPA-IgG. As shown in Table 1, this analysis, e.g. comparing ACPA IgA-positive to ACPA-IgA-negative patients in the presence of ACPA IgG, revealed no association of SE with ACPA-IgA, nor with ACPA-IgM or the AAPA isotypes (Table S10).

**Table 1:** Association of SE with AMPA isotypes in the EAC

	**SE neg n(%)**	**SE pos n(%)**	**OR (95% CI)**^**a**^	***p*-value**	**OR (95% CI)**^**a**^	***p*-value**
Healthy controls	674 (55.7)	537 (44.3)	1 (ref)			
ACPA-IgG -	154 (52.0)	142 (48.0)	1.12 (0.90–1.49)	0.23	1 (ref)	-
ACPA-IgG +	66 (20.9)	250 (79.2)	**4.75 (3.54–6.38)**	** < 0.001**	**4.11 (2.88–5.86)**	** < 0.001**
Healthy controls	674 (55.7)	537 (44.3)	1 (ref)	**-**		
AAPA-IgG -	190 (44.7)	235 (55.3)	**1.55 (1.24–1.94)**	** < 0.001**	1 (ref)	-
AAPA-IgG +	48 (21.5)	175 (78.5)	**4.58 (3.26–6.21)**	** < 0.001**	**2.95 (2.03–4.28)**	** < 0.001**
Healthy controls	674 (55.7)	537 (44.3)	1 (ref)	**-**		
AAPA-IgG-ACPA-IgG +	25 (221)	88 (77.9)	**4.42 (2.79–6.99)**	** < 0.001**	1 (Ref)	-
AAPA-IgG + ACPA-IgG +	41 (20.1)	163 (79.9)	**4.99 (3.48–7.16)**	** < 0.001**	1.14 (0.64–1.98)	0.64
Healthy controls	674 (55.7)	537 (44.3)	1 (ref)	**-**		
ACPA-IgA-	200 (44)	259 (56)	**1.63 (1.31–2.02)**	** < 0.0001**		
ACPA-IgA +	38 (20)	150 (80)	**4.95 (3.41–7.20)**	** < 0.0001**		
ACPA-IgA-ACPA-IgG +	32 (23)	108 (77)	**4.24 (2.81–6.39)**	** < 0.0001**	1 (ref)	-
ACPA-IgA + ACPA-IgG +	34 (19)	143 (81)	**5.28 (3.57–7.80)**	** < 0.0001**	1.25 (0.72–2.15)	0.79
Healthy controls	674 (55.7)	537 (44.3)	1 (ref)	**-**		
AAPA-IgA-	205 (38.8)	323 (61.2)	**1.98 (1.61–2.44)**	** < 0.0001**		
AAPA-IgA +	33 (27.7)	86 (72.3)	**3.27 (2.16–4.97)**	** < 0.0001**		
AAPA-IgA-ACPA-IgG +	46 (21)	175 (79)	**4.77 (3.39–6.74)**	** < 0.0001**	1 (ref)	-
AAPA-IgA + ACPA-IgG +	20 (21)	76 (79)	**4.77 (2.88–7.91)**	** < 0.0001**	1.00 (0.56–1.81)	0.99

*EAC* Leiden early arthritis clinic, *AAPA* Anti-acetylated protein antibodies, *ACPA* Anti-citrullinated protein antibodies, *SE* Shared epitope, *OR* Odds ratio, *95%CI* 95% Confidence interval

^*^odds ratio of univariate logistic regression

**Fig. 5:**
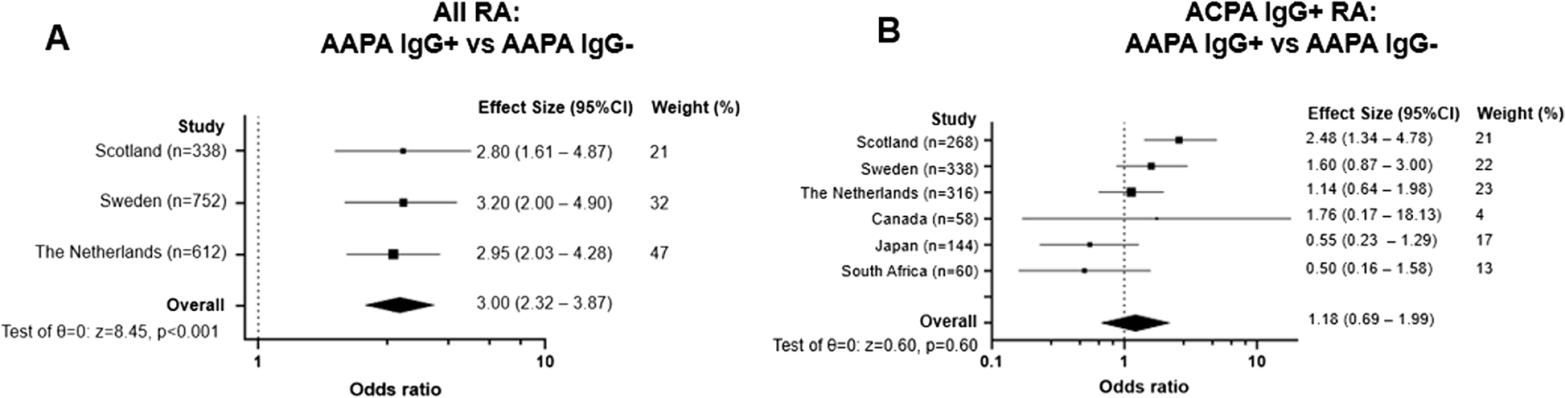
Meta-analysis combining study data with the SLR for the association of shared epitope with AAPA-IgG. **A**. meta-analysis of shared epitope with AAPA-IgG in all RA patients **B**. Meta-analysis of shared epitope in ACPA-IgG positive RA patients. AAPA: anti-acetylated protein antibodies, ACPA: anti-citrullinated protein antibodies, RA: rheumatoid arthritis; Scotland Scottish Early Rheumatoid Arthritis (SERA), Sweden: Epidemiological Investigations of RA (EIRA) [30] the Netherlands: Leiden early arthritis clinic (EAC), Canada [31], Japan [31], South Africa [31]

To further validate our findings, particularly the association (or lack thereof) between AAPA-IgG and SE, we conducted a meta-analysis. We obtained data from the SERA cohort and performed a systematic literature review (Figure S2). A meta-analysis combining these data with those from the EAC cohort revealed an association between SE and AAPA-IgG, with an odds ratio (OR) of 3.00 [2.32–3.87] (Fig. 5A). Given that AAPA-IgG is almost exclusively present in ACPA-IgG-positive patients, we then focused on this subgroup. We extended the meta-analysis by incorporating three additional, smaller cohorts that reported data exclusively within ACPA-IgG-positive patients. In the ACPA-positive subgroup, no significant association was found between SE and AAPA-IgG (OR 1.18 [0.69–1.99], Fig. 5B), suggesting that the observed association in Fig. 5A can be attributed to the concurrent presence of ACPA-IgG.

Taken together, these results indicate that in rheumatoid arthritis, SE is specifically associated with ACPA-IgG and not with other ACPA isotypes or other AMPA (Figure S4B).

To perform a more comprehensive investigation of possible genetic associations with AAPA-IgG, we also examined all HLA-DRB1 alleles, the well-known PTPN22 polymorphism associated with RA and a polygenic risk score. In line with our findings regarding SE, this revealed no genetic associations with AAPA-IgG (Table S11 and Table S12).

### Interaction analysis

An interaction is known to exist between SE alleles and smoking in predisposing for ACPA [4]. Since the findings described above indicate that SE is associated with ACPA-IgG while smoking is predominantly associated with AMPA-IgA, we wondered whether the known interaction would only be present in patients harboring both ACPA-IgA and ACPA-IgG and investigated this in both the EAC and SERA. In patients who were ACPA-IgG positive, but ACPA-IgA negative, no interaction was found (Figure S6 A, Table S13). In ACPA double positive (IgG + IgA +) patients on the contrary, an interaction of smoking and SE was present in the EAC with an AP 0.52 [0.23–0.81] and an S of 2.39 [1.15–4.99] and in the SERA with an AP 0.59 (0.04–1.13) (Table S13). Further analysis comparing ACPA IgA + IgG + patients to ACPA-IgG single-positive patients also revealed a significant interaction in the EAC, however in SERA no interaction was found (Table S13).

Similar effects were detected regarding AAPA. Here too, no interaction was present in AAPA IgA-negative patients, while a trend for interaction was found in the AAPA double-positive (IgG + IgA +) group (Figure S6 C and D, Table S13). To determine whether the effects for AAPA were independent of the presence of ACPA IgG, a separate analysis was performed in the ACPA-positive stratum, with analysis in the ACPA-negative stratum being impossible due to the absence of AAPA in those patients. This revealed no interaction between SE and smoking for AAPA in the presence of ACPA (Figure S6E, Table S13).

Together, these data show that the well-known interaction between smoking and SE is only present in ACPA-IgG positive patients when they are also ACPA-IgA positive (Fig. 4C, Table S13).

## Discussion

In this study, we have provided an in-depth investigation of the association of AMPA isotypes with smoking and genetic risk factors in RA. The results reveal that smoking is mainly associated with AMPA-IgA (ACPA and AAPA). With regard to genetic risk factors (SE, other HLA-DRB1 alleles and PTPN22) we only found associations with ACPA-IgG. Finally, interaction between smoking and SE was present in RA patients that were positive for both ACPA-IgA and ACPA-IgG (Fig. 4). These data are highly relevant for the pathophysiological understanding of RA since the association of smoking with AMPA-IgA further points to the importance of mucosal immune responses in the mechanisms underlying this disease.

Briefly, the mucosal origins hypothesis postulates that mucosal sites are strongly involved in development of RA-associated autoimmunity. Our data support this hypothesis since we observed that smoking is predominantly associated with IgA-ACPA and IgA-AAPA. This is in line with and extends previous observations by Svärd et al. to now include several different AMPA and a meta-analysis of the literature and unpublished data [29, 32]. The present finding that autoantibody-(IgA)-positive RA is particularly associated with current smoking is also compatible with a mucosal origin; with a serum half-life of on average four to seven days, it appears very plausible that an ongoing mucosal stimulus, such as current/continued smoking would be required to sustain IgA-production coming from mucosal sites. For anti-CarP we found a trend for an association between smoking and IgA which was not significant, possibly due to a lack of power since anti-CarP isotypes were measured in a smaller number of patients. Previously, we and others have described an association of smoking with RF, while in this study we only found an association with AMPA-IgA and not with RF specifically [6, 7, 33]. The explanation for these different observations could be that in the previous studies AMPA-IgA were not taken into account, since in the current analysis, we did also find an association of smoking with RF-IgA and RF-IgM, however after correcting for ACPA-IgA, the association with RF was lost.

The exact mechanisms linking smoking to AMPA-IgA, and consequently to possible mucosal immune responses, have yet to be fully understood. In addition to affecting the local abundance of PTM-antigens in the mucosa, smoking could also exert other effects on the immune system. As described in a recent publication, smoking increases the adaptive immune response of current and past smokers through epigenetic imprinting, and also increases the innate inflammatory response after bacterial stimulation [34].

Further research will also need to determine to what extent the development of ACPA-IgA occurs specifically via smoking in the lungs or can also develop via different stimuli and at other mucosal sites. Air pollution and silica exposure, known to be associated with RA, may be other contributing factors [35]. Another mucosal site possibly involved in the pathogenesis of RA is the oral cavity. ACPA have been detected in the saliva of autoantibody-positive RA patients, indicating localized production [36]. Furthermore, there are data suggesting a higher risk of RA development in patients with periodontitis associated with P. gingivalis [37]. Finally, the intestinal microbiome may play a role in RA, with indications that the microbiome of RA patients is disrupted and can be partially restored under treatment [38]. Whether the intestine may also be a site propagating or sustaining AMPA responses remains to be investigated.

Regarding the genetic predisposition to RA, we found that SE is exclusively linked to ACPA-IgG and not to other isotypes or AAPA. A previous report also did not observe an association between SE and AAPA-isotypes [30]. We have now added a meta-analysis of the association of SE with AAPA-IgG to the existing literature, which confirms that the SE alleles are not associated with AAPA-IgG. Previously, another AMPA present in RA patients: anti-CarP, was also found not to be associated with SE [39]. The unique association of ACPA-IgG with SE in contrast to other AMPAs reinforces the concept that AMPAs, despite being partly cross-reactive, are nonetheless distinct antibody responses with individual underlying predisposing factors [13]. This is in line with a previous publication describing a specific association of HLA B alleles with anti-CarP, in contrast to the HLA DR alleles associated with ACPA [40].

An interaction was observed between smoking and SE, but this interaction was only present in patients positive for both ACPA-IgA and ACPA-IgG. The possible explanation for this observed interaction pattern is that smoking appears to be linked predominantly with ACPA-IgA, while SE is associated with ACPA-IgG. These specific associations also suggest that different features of the ACPA response (i.e. IgA and IgG) might have different clonal or anatomical origins. Considering the order in which isotype switching occurs, and that ACPA IgA-reactivity is limited to ACPA IgG-positive patients, another scenario (besides the mucosal origins hypothesis) could be that an original systemic IgG response may undergo isotype switching to IgA upon exposure to e.g. cigarette smoke in the lungs. Further investigations delineating clonal relatedness between AMPA-IgG and IgA, for example by antibody sequencing, will be required to shed more light on this [41].

This study has several limitations. With regard to smoking in the Leiden EAC, for most patients only data on current smoking versus former/never smoking were available. However, since sub-analysis in the EAC, SERA and tREACH showed that the effect of smoking is strongest in current smokers, we do not expect that this definition of smoking has distorted the outcome. Moreover, we conducted a sub-analysis of the meta-analysis of smoking ever versus never with ACPA, which revealed the same results. In the sub-analyses in which the association of smoking and ACPA isotypes was corrected for autoantibody levels, the high collinearity between ACPA-IgA-levels and ACPA-IgG-levels prohibited correcting one for the other. Therefore, this specific sub-analysis was corrected for ACPA isotype positivity instead of for levels thus still allowing for a valid estimation of the individual associations. Moreover, our meta-analysis showed similar results. The meta-analysis of the association of SE with AAPA-IgG included cohorts with different ethnicities and symptom durations of RA, which may have played a role in the variation in effect sizes [30, 31]. Despite minor nuances per ethnicity of the exact contributing HLA-DRB1 alleles, the overall association of SE with RA has been described in many different ethnic backgrounds [42–44]. Therefore, the inclusion of diverse populations in the meta-analysis can also be viewed as a strength rather than a weakness, in the sense that it provides a truly global estimate of the association between SE alleles and AAPA.

Strengths of the current study are that we measured multiple AMPA, with assays that included robust controls utilizing non-modified peptides. Additionally, AAPA and ACPA were measured on the same backbone, indicating that the differences in associations of these AMPA with genetic and environmental risk factors can be attributed solely to differences in recognition of a specific post-translational modification and not to variation of the backbone. Secondly, for the analysis we made use of data from well-characterized EAC with extensive genetic data available, which we complemented with meta-analysis based on existing literature. For this meta-analysis, we gathered additional, previously unpublished data from several different cohorts. This validation significantly improved the reliability of our findings.

## Conclusions

This comprehensive analysis of associations between known risk factors and autoantibodies in RA indicates that smoking is predominantly associated with AMPA-IgA, while SE is associated with ACPA-IgG. The genetic-environment interaction between smoking and SE is found solely in patients who are positive for both ACPA-IgA and ACPA-IgG. These data provide a pivotal refinement of existing knowledge regarding risk factor associations for RA and lend novel support for the hypothesis that smoking may exert its effect on RA by the induction of local (auto)immune responses at mucosal sites.

## Supplementary Information

Supplementary Material 1.

## Data Availability

The datasets from Leiden generated during and/or analyzed during the current study are available from the corresponding author on reasonable request. The datasets from SERA are available for use subject to approval by the Scottish Early Rheumatoid Arthritis Access Committee.

## Abbreviations

AAPA: Anti-acetylated protein antibodies
ACPA: Anti-citrullinated protein antibodies
AMPA: Anti-modified protein antibodies
Anti-CarP: Anti-carbamylated protein antibodies
AP: Attributable proportion due to interaction
BAL: Broncho-alveolar lavage
EAC: Leiden early arthritis clinic
OR: Odds ratio
PTPN22: Protein tyrosine phosphatase non-receptor type 22
RA: Rheumatoid arthritis
RERI: Relative excess risk due to interaction
RF: Reumatoid factor
S: Synergy index
SE: HLA-DRB1 shared epitope alleles
SERA: Scottish Early Rheumatoid Arthritis
tREACH: Treatment in the Rotterdam Early Arthritis Cohort
