# Supplementary material for: Smoking as a risk factor for rheumatoid arthritis: predominant association with IgA autoantibodies – comprehensive analysis of anti-modified protein antibodies with smoking and genetic risk factors in rheumatoid arthritis
Source: Arthritis Res Ther. 2025 May 8;27:101. doi: 10.1186/s13075-025-03543-6 (PMC12060357; doi:10.1186/s13075-025-03543-6)
Supplement: Supplementary file 1 — Supplementary Material 1. [file 13075_2025_3543_MOESM1_ESM.docx]

**Content list**

| Supplementary methods |  | 2 |
| --- | --- | --- |
|  | Population | 2 |
|  | Detection of autoantibodies | 2 |
|  | Literature search for meta-analysis | 3 |
| Supplementary results |  | 4 |
|  | Rheumatoid factor (RF) and smoking in the Leiden EAC | 4 |
|  | Sub meta-analysis of smoking | 4 |
| Supplementary tables |  | 5 |
|  | Table S1. Baseline characteristics of rheumatoid arthritis patients in the EAC cohort | 5 |
|  | Table S2. Prevalence of AAPA and ACPA isotypes in the EAC (n (%) | 6 |
|  | Table S3. Association of smoking with autoantibodies in rheumatoid arthritis patients | 7 |
|  | Table S4. Association of smoking with ACPA-IgA & -IgG, AAPA-IgA & -IgG, AAPA-IgA & ACPA-IgA, and RF-IgA & -IgM | 8 |
|  | Table S5. Association of smoking with ACPA, AAPA, CarP and RF isotypes corrected for levels of autoantibodies | 9 |
|  | Table S6. Association of smoking with ACPA-IgA with RF-IgA & -IgM | 10 |
|  | Table S7. Association of smoking with ACPA-IgA with RF-IgA & -IgM corrected for levels of autoantibodies | 10 |
|  | Table S8. Association of current and past smoking with AMPA and RF isotypes | 11 |
|  | Table S9. Association of the number of copies of HLA SE with ACPA | 12 |
|  | Table S10. Association of HLA SE with AAPA-IgA, AAPA-IgM, ACPA-IgA and ACPA-IgM | 12 |
|  | Table S11. Association of HLA SE, PTPN22 and HLA-DRB1 genes with AAPA-IgG | 13 |
|  | Table S12. Association of polygenic risk score (PRS) with AAPA-IgG | 15 |
|  | Table S13. Interaction analysis of shared epitope and smoking with anti-acetylated protein antibodies and anti-citrullinated antibodies | 16 |
| Supplementary figures |  |  |
|  | Figure S1. flowchart systematic literature review smoking and ACPA isotypes | 18 |
|  | Figure S2. flowchart systematic literature review shared epitope and AAPA-IgG | 19 |
|  | Figure S3. Association of smoking corrected for autoantibody levels | 20 |
|  | Figure S4. Sub meta-analysis combining data from the EAC, SERA and tREACH of smoking with ACPA-IgG and ACPA-IgA | 21 |
|  | Figure S5. Sub meta-analysis combining data from the EAC, SERA and tREACH with the SLR of smoking ever with ACPA-IgG and ACPA-IgA | 22 |
|  | Figure S6. Interaction of SE and smoking with AAPA | 23 |
| References |  | 24 |

**Supplementary methods**

Population

Three different cohorts were investigated, originating from the Netherlands and Scotland, UK.

Data from the Dutch patients originated from the Leiden early arthritis clinic in which patients with recent onset of arthritis (duration less than two years) are included [1]. For the present study, data were included from 618 RA patients who fulfilled the American College of Rheumatology 1987 revised criteria for the classification of RA within 1 year of follow up. Dutch controls for HLA-DRB1 genetic analysis were randomly selected from the collection of the section of Immunogenetics and Transplantation Immunology from the LUMC. These controls were unrelated to the patients from the EAC and had no rheumatic diagnosis.

Data from the second Dutch cohort originated from the “treatment in the Rotterdam Early Arthritis Cohort” (tREACH) [2, 3]. The original tREACH was a multicenter stratified single-blinded RCT including early arthritis patients with at least one swollen joint and a symptom duration of <1 year [2, 3]. For the current study, 317 patients who fulfilled the 1987 or 2010 RA criteria were included of whom baseline data on smoking status and ACPA-IgA and ACPA- IgG were available.

Data from the Scottish patients originated from the Scottish Early Rheumatoid Arthritis (SERA)-inception cohort. The SERA cohort recruited patients with new-onset RA or undifferentiated arthritis who had at least one swollen joint from 20 hospitals across Scotland [4]. For the current study 369 RA patients fulfilling the RA 2010 criteria were included. The non-RA controls for genetic analysis were selected from the Generation Scotland biobank [5].

The protocol of each cohort was approved by the relevant local ethics committee and all participants provided written informed consent.

Detection of autoantibodies

All antibodies in the EAC were measured in baseline serum samples. Antibodies against ACPA-IgG were measured by the CCP2 Enzyme-linked immune sorbent assay (ELISA) (Immunoscan RA Mark 2; Eurodiagnostica), the cutoff was set at a value above 25 U/mL, according to the manufacturer’s instructions. ACPA IgG and IgM were measured by in-house CCP2 ELISA [6].

AAPA isotypes were detected by an inhouse ELISA with the following protocol: Nunc Maxisorp plates (Thermo Scientific) were coated with streptavidin (Invitrogen) overnight at 4 ˚ Celsius (C). Following washing in buffered sodium chloride (PBS) containing 0.05% tween (Sigma), the plates were incubated for one hour at 37˚ C with 50 µl of biotinylated peptide with the same configuration as CCP2 but an acetyl at the position of citrulline at a concentration of 1 µg/ml. After another washing step, 50 µl serum at a 1/50 dilution in PBS/1% bovine serum albumin (Sigma)/0.05% tween (PBT) was added to the plate and incubated over night at 4˚ C. As a standard, serial dilution of pooled positive sera was used. The wells were washed and bound antibodies were detected using horseradish peroxidase (HRP)-labelled goat-anti-human IgG (Dako) at a concentration of 1/5000 in PBT and incubated for one hour at 37˚ C. Following washing, the presence of anti-acetylated protein antibodies was detected using 2,2'-azino-bis-3-ethylbenzthiazoline-6-sulphonic-acid substrate (ABTS; Sigma) as substrate. The plate was developed for one hour and read out at a wavelength of 415 nm. As a control for acetyl lysine specificity, plates were incubated with norleucine with the same protocol as described above. Norleucine was selected as control peptide instead of lysine since lysine is positively charged at a normal pH whereas acetyl lysine is neutrally charged. Norleucine is neutrally charged and besides being one carbon group shorter than lysine, it has the same configuration as lysine. The cutoff for positivity was set as the mean plus two times the standard deviation of 80 controls. A sample was considered positive if it was above the cutoff and was 0.1 optical density higher on the acetylated peptide than on the control peptide.

IgM-RF was measured by a commercial ELISA and the cutoff was set as recommended by the manufacturer. RF-IgA was measured with an inhouse ELISA. Briefly, nunc Maxisorp plates (Thermo Scientific) were coated with 5 µg/ml rabbit IgG (Sigma) overnight at room temperature. Following washing in buffered sodium chloride (PBS) containing 0.05% tween (Sigma), the plates were blocked by adding 100 µg /well of PBS/1%BSA for one hour at 37˚ C. After washing, a commercial standard (N/T Rheumatology control SL/2; Siemens) and samples diluted 1:250 were added to the plates and incubated for one hour at 37˚ C. The wells were washed and bound antibodies were detected using (HRP)-labelled goat-anti-human IgA (Invitrogen) at a concentration of 1/5000 in PBT and incubated for one hour at 37˚ C. Following washing, the presence of RF-IgA was detected using 2,2'-azino-bis-3-ethylbenzthiazoline-6-sulphonic-acid substrate (ABTS; Sigma) as substrate. The plate was developed for one hour and read out at a wavelength of 415 nm.

The cut-off for autoantibody detection was determined based on the manufacturer's guidelines or, for in-house ELISAs, established to ensure comparable specificity across all autoantibodies. A maximum of 2.5% of healthy controls tested positive, corresponding to a specificity of 97.5%.

In the tREACH antibodies were measured in baseline serum samples (collected at disease onset). The presence of autoantibody isotypes ACPA-IgG, ACPA-IgA, RF-IgM, and RF-IgA was determined by automated fluorescence enzyme-immuno assay (FEIA) using the Phadia250 EliA™ platform (Thermo Fisher Scientific, Freiburg, Germany). Cut-off levels for autoantibody positivity were set according to the manufacturer’s instruction, with the cut-off levels for both ACPA-IgG and ACPA-IgA positivity being ≥7 U/ml.

In the SERA cohort, AMPAs were measured at baseline in plasma samples by the standard protocol for the Orgentec Diagnostika ELISA as described previously [7]. The cut-off levels for AMPA positivity being ≥25 U/ml.

Literature search for meta-analysis

A literature search was performed to investigate associations between smoking and anti-citrullinated protein antibodies (ACPA) isotypes in rheumatoid arthritis (RA) patients. See figure S1 for a flowchart of the article selection. PubMed was searched until April 2023 with the following search strategy:

("Arthritis, Rheumatoid"[Majr] OR "Rheumatoid Arthritis"[ti]) AND ("Smoking"[Mesh] OR "Smoking"[tw] OR "Smokes"[tw] OR "Smoked"[tw]) AND ("ACPA"[tiab] OR "AAPA"[tiab] OR "anti-citrullinated protein antibod*"[tw] OR "citrullinated protein antibod*"[tw] OR "anti citrullinated protein antibod*"[tw] OR "anti-citrullinated peptide antibodies"[tw] OR "anti citrullinated peptide antibodies"[tw] OR "anti-citrullinated peptide autoantibodies"[tw] OR "anti citrullinated peptide autoantibodies"[tw] OR "anti-acetylated protein antibod*"[tw] OR "Acetylation"[Mesh] OR "acetylation*"[tw] OR "Cyclic Citrullinated Peptides antibodies"[tiab:~1] OR "Cyclic Citrullinated Peptide antibody"[tiab:~1] OR "anti-CCP"[tw] OR "anti-modified protein antibod*"[tw] OR "AMPA"[tiab] OR "rheumatoid factor"[tw] OR "Anti-Citrullinated Protein Antibodies"[Mesh]) AND ("IgA"[tw] OR "IgA1"[tw] OR "Immunoglobulin A"[Mesh] OR "Immunoglobulin A"[tw])

After removal of reviews, duplicates and articles that did not apply to our research question (based on title and abstract screening), two articles including three cohorts remained[8, 9].

A literature search was performed to investigate associations between HLA-DRB1 shared epitope (SE) and anti-acetylated protein antibodies (AAPA) in rheumatoid arthritis (RA) patients. See figure S2 for a flowchart of the article selection. PubMed was searched until April 2023 with the following search strategy:

("Arthritis, Rheumatoid"[Mesh] OR "Rheumatoid Arthritis"[tw]) AND ("Epitopes"[Mesh] OR "shared epitope*"[tw] OR "HLA-DR Antigens"[Mesh] OR "HLA-DR*"[tw]) AND ("AAPA"[tiab] OR "anti-acetylated protein antibodies"[tw] OR "anti-modified protein antibodies"[tw] OR "AMPA"[tiab] OR "Acetylation"[Mesh] OR "acetylation*"[tw])

After removal of reviews, duplicates and articles that did not apply to our research question (based on title and abstract screening), two articles remained[10, 11].

**Analysis**

Rheumatoid factor (RF) and smoking in the Leiden EAC

For RF, the findings were slightly different: the association of smoking with both RF-IgM and -IgA was lost when correcting for levels of the other isotype (correcting RF-IgM for RF-IgA and RF-IgA for RF-IgM) (Figure S3D and Table S5). In light of the distinct association between smoking and ACPA-IgA described above, and knowing that ACPA-IgA is associated with RF, we further dissected the connection between RF IgM and IgA by also taking ACPA-IgA into account. As shown in Table S6, there was again only an association of smoking with RF-IgM or RF-IgA in the presence of ACPA-IgA, i.e. in the double positive groups. After correcting the association of smoking with RF-IgM and RF-IgA for ACPA-IgA levels, the association between smoking and RF-IgM and RF-IgA was lost (Table S7). However, when ACPA-IgA was corrected for RF-IgM levels or RF-IgA levels, smoking was still associated with ACPA-IgA, although in the latter analysis this did not quite achieve statistical significance(Figure S3D, Table S7). This indicated that the previously found association of smoking with RF-IgM and RF-IgA is caused by confounding due to ACPA-IgA.

Sub meta-analysis of smoking

A sub-meta-analysis was performed including only the cohorts with available data on current versus never/past smoking which revealed similar results (Figure S4). Finally, we conducted a meta-analysis of all cohorts including data extracted from the literature with smoking defined as ever versus never. The data now also showed a small, yet significant association with ACPA-IgG single positive patients (OR 1.25 [1.02-1.52]) (Figure S5A). However the strongest association was again found in ACPA double positive patients (IgG+ IgA+) with a significant additive effect of the simultaneous presence of ACPA IgA and IgG compared to ACPA-IgG alone(Figure S5D).Taken together, these data indicate that smoking is predominantly associated with AMPA-IgA.

**Supplementary tables**

**Table S1.** Baseline characteristics of rheumatoid arthritis patients in the EAC, SERA and tREACH cohort

| **Characteristic** | **EAC (n=618) *** | **SERA* (n=351)** | **tREACH (n=316)** |
| --- | --- | --- | --- |
| Age (years), mean +-SD | 57.4 +/- 15.7 | 58.7 +/- 13.4 | 54.2 (14.0) |
| Female, n (%) | 409 (66.2) | 233 (66.4) | 222 (66.3) |
| BMI, mean +-SD | 25.9 +/- 3.9 | 28.0 +/- 5.4 | 26.4 (4.9) |
| Fam history of RA, n (%) | 152 (25.3) |  | 125 (37.3) |
| Smoking (current), n (%) | 154 (24.9) | 87 (24.8) | 97 (30.6) |
| Smoking (ever), n (%) data >2002 | 138 (60.0) | 221 (63.0) | 212 (67.1) |
| Symptom duration (weeks), median (IQR) | 18 (9 -35) | 28 (19 - 54) | 21 (13-30) |
| ESR (mm/hour), median (IQR) | 34 (19 - 54) | 26 (14 - 44) | 20 (11-37) |
| CRP (mg/L), median (IQR) | 18 (8 - 40) | 11.5 (4 - 32) | 7 (4-20) |
| SJC (in 28 joints), median (IQR) | 6 (3 - 11) | 6 (3 - 10) | 6 (3-10) |
| TJC (in 28 joints), median (IQR) | 8 (4 - 14) | 8 (3 - 15) | 6 (3-10) |
| VAS (0-100), median (IQR) | 42 (20 - 58) | 51 (31 - 71) | 52 (31-68) |
| DAS-28, mean +-SD | 5.15 +-1.26 | 5.14 +/- 1.35 | 4.78 (1.17) |
| DAS-28-CRP, mean +-SD |  | 4.90 +/- 1.23 |  |
| HAQ, median (IQR) | 1.00 (0.62 - 1.62) | 1.25 (0.63 - 1.88) | 1.00 (0.38-1.50) |
| RF-positive, n (%) | 348 (56.5) | 199 (56.7) | 196 (58.5) |
| ACPA IgG-positive, n (%) | 317 (51.3) | 279 (79.5) | 177 (52.8) |
| AAPA IgG-positive, n (%) | 208 (33.7) | 123 (35.0) | - |
| Anti-CarP IgG-positive, n (%) | 270 (44.1) | 167 (47.6) | - |
| HLA-DRB1 shared epitope positive, n (%) | 410 (64.0) | 238 (70.4) | - |
| HLA-DRB1 shared epitope homozygote | 68 (10.5) | 19 (5.6) | - |

* numbers might slightly differ per outcome due to missing data of some of the outcomes

EAC: Leiden early arthritis clinic, tREACH: treatment in the Rotterdam Early Arthritis Cohort, SERA: Scottish Early Rheumatoid Arthritis BMI: body-mass index, ESR: Erythrocyte Sedimentation Rate, CRP:  C-reactive protein, SJC: swollen joint count, TJC: tender joint count, VAS: Visual Analogue Scale, HAQ: Health Assessment Questionnaire-Disability Index, SHS: Sharp-van der Heijde score, RF: rheumatoid factor, ACPA: anti-citrullinated protein antibodies, anti-CarP: anti-carbamylated protein antibodies, AAPA: anti-acetylated protein antibodies

**Table S2.** Prevalence of AAPA and ACPA isotypes in the EAC

|  | **ACPA, n (%)** | **AAPA, n (%)** |
| --- | --- | --- |
| IgG- IgA- IgM- | 299 (48) | 334 (54) |
| **IgG+** IgA- IgM- | 115 (19) | 100 (16) |
| IgG- **IgA+** IgM- | 1 (0) | 15 (2) |
| IgG- IgA- **IgM+** | 0 (0) | 52 (8) |
| **IgG+ IgA+** IgM- | 41 (7) | 31 (5) |
| **IgG+** IgA- **IgM+** | 25 (4) | 22 (4) |
| IgG- **IgA+ IgM+** | 0 (0) | 9 (1) |
| **IgG+ IgA+ IgM+** | 136 (22) | 55 (9) |

ACPA: anti-citrullinated protein antibodies, AAPA: anti-acetylated protein antibodies, EAC: Leiden early arthritis clinic

**Table S3.** Association of smoking* with autoantibodies in rheumatoid arthritis patients in the EAC

| **Antibody** | **Odds ratio (95%CI)^** | **p-value** |
| --- | --- | --- |
| ACPA IgG | **1.52 (1.05 - 2.20)** | **0.03** |
| ACPA IgA | **1.97 (1.34 - 2.89)** | **0.001** |
| AAPA IgG | 1.41 (0.97 - 2.06) | 0.07 |
| AAPA IgA | **2.41 (1.56 - 3.73)** | **<0.0001** |
| CarP IgG | 1.32 (0.81 - 2.17) | 0.27 |
| CarP IgA | 1.44 (0.88 - 2.38) | 0.15 |
| RF IgM | **1.66 (1.14 - 2.42)** | **0.01** |
| RF IgA | **1.49 (1.01 - 2.21)** | **0.04** |

*smoking was defined as current versus never/past. ^odds ratio of univariate logistic regression. EAC: Leiden early arthritis clinic AAPA: anti-acetylated protein antibodies, ACPA: anti-citrullinated protein antibodies, RF: rheumatoid factor, CarP: anti-carbamylated protein antibodies, OR: odds ratio, 95% CI: 95% confidence interval

**.**

**Table S4.** Association of smoking* with ACPA-IgA & -IgG, AAPA-IgA & -IgG, AAPA-IgA & ACPA-IgA, and RF-IgA & -IgM in the EAC

| **EAC** | | | | | | | | |
| --- | --- | --- | --- | --- | --- | --- | --- | --- |
|  | **smoking neg n(%)** | **Smoking pos n(%)** | **OR (95% CI)^** | **p-value** | **OR (95% CI)** | **p-value** | **OR (95% CI) ^** | **p-value** |
| ACPA IgA- ACPA IgG - | 237 (79) | 62 (21) | 1 (ref) | - |  |  |  |  |
| **ACPA IgA+** ACPA IgG- | 1 (100) | 0 (0) |  |  |  |  |  |  |
| ACPA IgA- **ACPA IgG+** | 110 (79) | 30 (21) | 1.04 (0.64 - 1.70) | - |  |  | 1 (ref) | - |
| **ACPA IgA+ ACPA IgG+** | 116 (66) | 61 (34) | **2.01 (1.32 - 3.05)** | **0.001** |  |  | **1.93 (1.16 - 3.21)** | **0.011** |
|  |  |  |  |  |  |  |  |  |
|  | **smoking neg n(%)** | **Smoking pos n(%)** | **OR (95% CI) ^** | **p-value** | **OR (95% CI)** | **p-value** | **OR (95% CI) ^** | **p-value** |
| AAPA IgA- AAPA IgG- | 299 (77) | 87 (23) | 1 (ref) | - |  |  |  |  |
| **AAPA IgA+** AAPA IgG- | 18 (75) | 6 (25) | 1.15 (0.44 - 2.97) | 0.78 | 1 (ref) | - |  |  |
| AAPA IgA- **AAPA IgG+** | 99 (81) | 23 (19) | 0.80 (0.48 - 1.33) | 0.40 |  |  | 1 (ref) | - |
| **AAPA IgA+ AAPA IgG+** | 48 (56) | 38 (44) | **2.72 (1.67 - 4.43)** | **<0.0001** | 2.38 (0.86 - 6.57) | 0.10 | **3.41 (1.83 - 6.35)** | **<0.0001** |
|  |  |  |  |  |  |  |  |  |
|  | **smoking neg n(%)** | **Smoking pos n(%)** | **OR (95% CI) ^** | **p-value** | **OR (95% CI)** | **p-value** | **OR (95% CI) ^** | **p-value** |
| CarP IgA- CarP IgG- | 93 (74) | 32 (26) | 1 (ref) | - |  |  |  |  |
| **CarP IgA+** CarP IgG- | 31 (74) | 11 (26) | 1.03 (0.46 - 2.29) | 0.94 | 1 (ref) | - |  |  |
| CarP IgA- **CarP IgG+** | 45 (75) | 15 (25) | 0.97 (0.48 - 1.97) | 0.93 |  |  | 1 (ref) | - |
| **CarP IgA+ CarP IgG+** | 53 (64) | 30 (36) | 1.64 (0.90 - 3.00) | 0.10 | 1.60 (0.70 - 3.62) | 0.27 | 1.70 (0.81 - 3.55) | 0.15 |
|  |  |  |  |  |  |  |  |  |
|  | **smoking neg n(%)** | **Smoking pos n(%)** | **OR (95% CI) ^** | **p-value** | **OR (95% CI)** | **p-value** | **OR (95% CI) ^** | **p-value** |
| RF IgA- RF IgM- | 145 (79) | 38 (21) | 1 (ref) | - |  |  |  |  |
| **RF IgA+** RF IgM- | 70 (82) | 15 (18) | 0.82 (0.42 - 1.59) | 0.55 | 1 (ref) | - |  |  |
| RF IgA- **RF IgM+** | 39 (81) | 9 (19) | 0.88 (0.39 - 1.98) | 0.76 |  |  | 1 (ref) | - |
| **RF IgA+ RF IgM+** | 208 (70) | 91 (30) | **1.67 (1.08 - 2.58)** | **0.02** | **2.04 (1.11 - 3.76)** | **0.02** | 1.89 (0.88 - 4.08) | 0.10 |

*smoking was defined as current versus never/past. ^odds ratio of univariate logistic regression. CarP: smaller numbers of patients are included in this analysis since CarP-IgA was measured in a subgroup of patients from EAC AAPA: anti-acetylated protein antibodies, ACPA: anti-citrullinated protein antibodies, CarP: anti-carbamylated protein antibodies, RF: rheumatoid factor, OR: odds ratio, 95% CI: 95% confidence interval, EAC: Leiden early arthritis clinic

**Table S5.** Association of smoking^ with ACPA, AAPA, CarP and RF isotypes corrected for levels of autoantibodies in the EAC

| Smoking | OR (95% CI) | p-value | OR (95% CI) | p-value |
| --- | --- | --- | --- | --- |
|  | Univariate | | multivariable with as covariate | |
|  |  | | ACPA-IgG positivity | |
| ACPA-IgA* | **1.97 (1.34 - 2.89)** | **0.001** | **1.89 (1.14 - 3.12)** | **0.014** |
|  |  | | ACPA-IgA positivity | |
| ACPA-IgG* | **1.52 (1.05 - 2.20)** | **0.03** | 1.06 (0.65 - 1.73) | 0.80 |
|  | | | | |
|  |  | | AAPA-IgG levels | |
| AAPA-IgA* | **2.41 (1.56 - 3.73)** | **<0.0001** | **2.30 (1.35 - 3.94)** | **0.002** |
|  |  | | AAPA-IgA levels | |
| AAPA-IgG* | 1.41 (0.97 - 2.06) | 0.07 | 1.14 (0.75 - 1.73) | 0.53 |
|  | | | | |
|  |  | | CarP-IgG levels | |
| CarP-IgA* | 1.44 (0.88 - 2.38) | 0.15 | 1.19 (0.70 - 2.04) | 0.53 |
|  |  | | CarP-IgA levels | |
| CarP-IgG* | 1.32 (0.81 - 2.17) | 0.27 | 1.10 (0.64 - 1.90) | 0.72 |
|  | | | | |
|  |  | | RF-IgM levels | |
| RF-IgA* | **1.49 (1.01 - 2.21)** | **0.04** | 1.16 (0.74 - 1.83) | 0.52 |
|  |  | | RF-IgA levels | |
| RF-IgM* | **1.66 (1.14 - 2.42)** | **0.01** | 1.02 (0.63 - 1.64) | 0.95 |

*the autoantibody negative group is the reference group. ^smoking was defined as current versus never/past EAC: Leiden early arthritis clinic, AAPA: anti-acetylated protein antibodies, ACPA: anti-citrullinated protein antibodies, CarP: anti-carbamylated protein antibodies, RF: rheumatoid factor, OR: odds ratio, 95% CI: 95% confidence interval

**Table S6.** Association of smoking with ACPA-IgA with RF-IgA & -IgM in the EAC

|  | **smoking neg n(%)** | **Smoking pos n(%)** | **OR (95% CI)*** | **p-value** | **OR (95% CI)*** | **p-value** | **OR (95% CI)*** | **p-value** |
| --- | --- | --- | --- | --- | --- | --- | --- | --- |
| ACPA IgA- RF IgM- | 205 (81) | 49 (19) | 1 (ref) | - |  |  |  |  |
| **ACPA IgA+** RF IgM- | 10 (71) | 4 (29) | 1.67 (0.50 - 5.56) | 0.40 | 1 (ref) | - |  |  |
| ACPA IgA- **RF IgM+** | 140 (77) | 43 (24) | 1.29 (0.81 - 2.04) | 0.29 |  |  | 1 (ref) | - |
| **ACPA IgA+ RF IgM+** | 107 (64) | 57 (35) | **2.23 (1.42 - 3.49)** | **<0.0001** | 1.33 (0.40 - 4.44) | 0.64 | **1.73 (1.08 - 2.77)** | **0.02** |
|  |  |  |  |  |  |  |  |  |
|  | **smoking neg n(%)** | **Smoking pos n(%)** | **OR (95% CI)*** | **p-value** | **OR (95% CI)*** | **p-value** | **OR (95% CI)*** | **p-value** |
| ACPA IgA- RF IgA- | 173 (81) | 41 (19) | 1 (ref) | - |  |  |  |  |
| **ACPA IgA+** RF IgA- | 12 (67) | 6 (33) | 2.11 (0.75 - 5.95) | 0.16 | 1 (ref) | - |  |  |
| ACPA IgA- **RF IgA+** | 174 (77) | 51 (23) | 1.24 (0.78 - 1.96) | 0.37 |  |  | 1 (ref) | - |
| **ACPA IgA+ RF IgA+** | 105 (66) | 55 (24) | **2.21 (1.38 - 3.54)** | **0.001** | 1.05 (0.37 - 2.94) | 0.93 | **1.79 (1.14 - 2.81)** | **0.012** |

*odds ratio of univariate logistic regression. EAC: Leiden early arthritis clinic ACPA: anti-citrullinated protein antibodies, RF: rheumatoid factor, OR: odds ratio, 95% CI: 95% confidence interval

**Table S7.** Association of smoking of ACPA-IgA and RF-IgA & RF-IgM corrected for levels of autoantibodies in the EAC

| Smoking | OR (95% CI)^ | p-value | OR (95% CI)^ | p-value |
| --- | --- | --- | --- | --- |
|  | univariate | | multivariate with as covariate | |
|  |  |  | RF-IgM levels | |
| ACPA-IgA* | **1.97 (1.34 - 2.89)** | **0.001** | **1.64 (1.09 - 2.48)** | **0.02** |
|  |  |  | ACPA-IgA levels | |
| RF-IgM* | **1.66 (1.14 - 2.42)** | **0.01** | 1.10 (0.71 - 1.72) | 0.66 |
|  |  |  |  |  |
|  |  |  | RF-IgA levels |  |
| ACPA-IgA* | **1.97 (1.34 - 2.89)** | **0.001** | 1.45 (0.95 - 2.21) | 0.09 |
|  |  |  | ACPA-IgA levels |  |
| RF-IgA* | **1.66 (1.14 - 2.42)** | **0.01** | 1.01 (0.65 - 1.57) | 0.96 |

*the autoantibody negative group is the reference group. ^odds ratio of univariate logistic regression. EAC: Leiden early arthritis clinic, AAPA: anti-acetylated protein antibodies, ACPA: anti-citrullinated protein antibodies, CarP: anti-carbamylated protein antibodies, RF: rheumatoid factor, OR: odds ratio, 95% CI: 95% confidence interval

**Table S8.** Association of current and past smoking with AMPA and RF isotypes in the EAC, SERA and tREACH

| **EAC*** | | | | | | | | |
| --- | --- | --- | --- | --- | --- | --- | --- | --- |
|  | **Smoking ever versus smoking never** | | **Smoking current versus never/past** | | **Smoking current versus never** | | **Smoking past versus never** | |
| **Antibody** | **Odds ratio (95%CI)** | **p-value** | **Odds ratio (95%CI)** | **p-value** | **Odds ratio (95%CI)** | **p-value** | **Odds ratio (95%CI)** | **p-value** |
| ACPA IgG | 1.26 (0.74 - 2.14) | 0.39 | **2.27 (1.10 - 4.71)** | **0.03** | **2.28 (1.04 - 5.02)** | **0.04** | 1.01 (0.58 - 1.79) | 0.96 |
| ACPA IgA | 1.14 (0.62 - 2.08) | 0.67 | **2.08 (1.00 - 4.30)** | **0.05** | 1.96 (0.88 - 4.37) | 0.10 | 0.90 (0.46 - 1.74) | 0.75 |
| AAPA IgG | 0.82 (0.46 - 1.44) | 0.49 | 1.28 (0.62 - 2.65) | 0.50 | 1.09 (0.50 - 2.40) | 0.82 | 0.73 (0.40 - 1.34) | 0.31 |
| AAPA IgA | 1.19 (0.56 - 2.57) | 0.65 | 2.17 (0.92 - 5.14) | 0.08 | 2.07 (0.79 - 5.42) | 0.14 | 0.91 (0.39 - 2.14) | 0.83 |
| RF IgM | 1.21 (0.71 - 2.07) | 0.49 | 1.68 (0.80 - 3.53) | 0.17 | 1.73 (0.78 - 3.86) | 0.18 | 1.06 (0.60 - 1.89) | 0.84 |
| RF IgA | 1.42 (0.83 - 2.42) | 0.20 | 1.52 (0.76 - 3.07) | 0.24 | 1.76 (0.82 - 3.76) | 0.15 | 1.31 (0.74 - 2.32) | 0.35 |
| CarP IgG | 0.94 (0.55 - 1.61) | 0.83 | 1.40 (0.70 - 2.83) | 0.34 | 1.28 (0.60 - 2.73) | 0.53 | 0.83 (4.56 - 1.49) | 0.54 |
| CarP IgA | 1.04 (0.18 - 6.12) | 0.96 | n.a. | n.a. | n.a. | n.a. | 0.63 (0.09 - 4.22) | 0.63 |
| **SERA** | | | | | | | | |
|  | **Smoking ever versus smoking never** | | **Smoking current versus never/past** | | **Smoking current versus never** | | **Smoking past versus never** | |
| **Antibody** | **Odds ratio (95%CI)** | **p-value** | **Odds ratio (95%CI)** | **p-value** | **Odds ratio (95%CI)** | **p-value** | **Odds ratio (95%CI)** | **p-value** |
| ACPA IgG | **2.44 (1.44 - 4.15)** | **0.0009** | **3.73 (1.75 - 9.26)** | **0.0017** | **4.90 (2.19 - 12.51)** | **0.0003** | **1.78 (1.01 - 3.17)** | **0.0471** |
| ACPA IgA | **1.77 (1.07 - 2.98)** | **0.028** | **2.69 (1.61 - 4.51)** | **0.0002** | **2.96 (1.63 - 5.43)** | **0.0004** | 1.20 (0.67 - 2.15) | 0.54 |
| AAPA IgG | 0.93 (0.59 - 1.46) | 0.74 | 1.44 (0.87 - 2.36) | 0.15 | 1.25 (0.71 - 2.18) | 0.44 | 0.75 (0.45 - 1.26) | 0.28 |
| AAPA IgA | 2.35 (0.92 - 7.23) | 0.10 | **4.04 (1.74 - 9.57)** | **0.0012** | **4.39 (1.59 - 14.14)** | **0.0067** | 1.17 (0.34 - 4.16) | 0.80 |
| RF IgM | **1.79 (1.16 - 2.78)** | **0.0093** | **2.66 (1.58 - 4.61)** | **0.0003** | **3.05 (1.71 - 5.57)** | **0.0002** | 1.31 (0.81 - 2.13) | 0.27 |
| RF IgA | **1.64 (1.06 - 2.54)** | **0.027** | **2.33 (1.40 - 3.94)** | **0.0013** | **2.59 (1.48 - 4.63)** | **0.0011** | 1.24 (0.76 - 2.01) | 0.39 |
| CarP IgG | **1.47 (0.95 - 2.29)** | **0.082** | **1.60 (0.98 - 2.61)** | **0.061** | **1.81 (1.05 - 3.16)** | **0.033** | 1.29 (0.79 - 2.10) | 0.31 |
| CarP IgA | 2.17 (0.90 - 6.05) | 0.10 | **3.70 (1.66 - 8.32)** | **0.0013** | **3.96 (1.52 - 11.61)** | **0.0069** | 1.14 (0.37 - 3.63) | 0.82 |
| **tREACH** | | | | | | | | |
|  | **Smoking ever versus smoking never** | | **Smoking current versus never/past** | | **Smoking current versus never** | | **Smoking past versus never** | |
| **Antibody** | **Odds ratio (95%CI)** | **p-value** | **Odds ratio (95%CI)** | **p-value** | **Odds ratio (95%CI)** | **p-value** | **Odds ratio (95%CI)** | **p-value** |
| ACPA IgG | **1.81 (1.13-2.91)** | **0.014** | **2.10 (1.28-3.45)** | **0.003** | **2.53 (1.43-4.47)** | **0.001** | 1.39 (0.81-2.37) | 0.229 |
| ACPA IgA | **2.11 (1.20-3.72)** | **0.009** | **1.98 (1.19-3.30)** | **0.009** | **2.71 (1.43-5.11)** | **0.002** | 1.69 (0.90-3.18) | 0.104 |
| RF IgM | 0.98 (0.61-1.57) | 0.917 | 1.08 (0.67-1.76) | 0.747 | 1.04 (0.60-1.83) | 0.878 | 0.92 (0.54-1.57) | 0.761 |
| RF IgA | **1.84 (1.14-2.97)** | **0.013** | **2.33 (1.43-3.80)** | **0.001** | **2.70 (1.52-4.77)** | **0.001** | 1.34 (0.78-2.30 | 0.296 |

*Analyses from the EAC were performed in a subgroup of RA patients (n=243) of whom data on past, current and never smoking were available

n.a. not applicable (calculation not possible since zero smokers in anti-CarP-IgA negative group). All odds ratio are from univariate logistic regression. EAC: Leiden early arthritis clinic, SERA: Scottish Early Rheumatoid Arthritis, tREACH: treatment in the Rotterdam Early Arthritis Cohort AAPA: anti-acetylated protein antibodies, ACPA: anti-citrullinated protein antibodies, CarP: anti-carbamylated protein antibodies, RF: rheumatoid factor, OR: odds ratio, 95% CI: 95% confidence interval.

**Table S9**. Association of the number of copies of HLA SE with ACPA

|  | **SE 1 copy n(%)** | **SE 2 copies (%)** | **OR (95% CI)*** | **p-value** |
| --- | --- | --- | --- | --- |
| ACPA-IgG - | 122 (98.1) | 15 (10.6) | 1 (ref) | - |
| ACPA-IgG+ | 180 (78.6) | 49 (21.4) | **2.21 (1.19 – 4.13)** | **0.01** |

*odds ratio of univariate logistic regression

**Table S10.** Association of HLA SE with AAPA-IgA, AAPA-IgM, ACPA-IgA and ACPA-IgM in the EAC

|  | **All RA patients** | | | | | |
| --- | --- | --- | --- | --- | --- | --- |
| **Autoantibody status** | **SE neg n(%)** | **SE pos n(%)** | **OR (95% CI)*** | **p-value** | **OR (95% CI)*** | **p-value** |
| Healthy controls | 674 (55.7) | 537 (44.3) | 1 (ref) | **-** |  |  |
| ACPA-IgA- | 200 (44) | 259 (56) | **1.63 (1.31 - 2.02)** | **<0.0001** | 1 (ref) | **-** |
| ACPA-IgA+ | 38 (20) | 150 (80) | **4.95 (3.41 - 7.20)** | **<0.0001** | **3.05 (2.04 - 4.55)** | **<0.001** |
|  | | | | | | |
| ACPA-IgM- | 204 (42.7) | 274 (57.3) | **1.69 (1.36 - 2.09)** | **<0.0001** | 1 (ref) | **-** |
| ACPA-IgM+ | 34 (20.1) | 135 (79.9) | **4.98 (3.36 - 7.38)** | **<0.0001** | **2.96 (1.95 - 4.49)** | **<0.0001** |
|  |  |  |  |  |  |  |
| AAPA-IgA- | 205 (38.8) | 323 (61.2) | **1.98 (1.61 - 2.44)** | **<0.0001** | 1 (ref) | **-** |
| AAPA-IgA+ | 33 (27.7) | 86 (72.3) | **3.27 (2.16 - 4.97)** | **<0.0001** | **1.65 (1.07 - 2.56)** | **0.02** |
|  |  |  |  |  |  |  |
| AAPA-IgM- | 198 (39.3) | 306 (60.7) | **1.94 (1.57 - 2.40)** | **<0.0001** | 1 (ref) | **-** |
| AAPA-IgM+ | 40 (27.8) | 104 (72.2) | **3.26 (2.23 - 4.78)** | **<0.0001** | **1.68 (1.12 - 2.53)** | **0.012** |
|  | **ACPA-IgG neg RA patients** | | | | | |
| Healthy controls | 674 (55.7) | 537 (44.3) | 1 (ref) | **-** |  |  |
| ACPA-IgA- | 156 (52) | 143 (48) | 1.15 (0.98 - 1.48) | 0.28 | 1 (ref) | **-** |
| ACPA-IgA+ | 0 (0) | 1 (100) | - | - | Na | na |
|  | | | | | | |
| ACPA-IgM- | 156 (52) | 144 (48) | 1.16 (0.90 - 1.49) | 0.26 | 1 (ref) | **-** |
| ACPA-IgM+ | 0 (0) | 0 (0) | - | - | Na | na |
|  | | | | | | |
| AAPA-IgA- | 148 (52) | 138 (48) | 1.17 (0.90 - 1.52) | 0.21 | 1 (ref) | **-** |
| AAPA-IgA+ | 8 (57) | 6 (43) | 0.94 (0.32 - 2.73) | 0.91 | 0.80 (0.27 - 2.38) | 0.69 |
|  |  |  |  |  |  |  |
| AAPA-IgM- | 141 (53.4) | 123 (46.6) | 1.09 (0.84 - 1.43) | 0.51 | 1 (ref) | **-** |
| AAPA-IgM+ | 15 (40.6) | 22 (59.4) | 1.84 (0.95 - 3.58) | 0.07 | 1.68 (0.84 0 3.38) | 0.15 |
|  | **ACPA-IgG pos RA patients** | | | | | |
| Healthy controls | 674 (55.7) | 537 (44.3) | 1 (ref) | **-** |  |  |
| ACPA-IgA- | 32 (23) | 108 (77) | **4.24 (2.81 - 6.39)** | **<0.0001** | 1 (ref) | **-** |
| ACPA-IgA+ | 34 (19) | 143 (81) | **5.28 (3.57 - 7.80)** | **<0.0001** | 1.25 (0.72 - 2.15) | 0.79 |
|  | | | | | | |
| ACPA-IgM- | 36 (23.1) | 120 (76.9) | **4.18 (2.83 - 6.18)** | **<0.0001** | 1 (ref) | **-** |
| ACPA-IgM+ | 30 (18.6) | 131 (81.4) | **5.48 (3.63 - 8.28)** | **<0.0001** | 1.31 (0.76 - 2.26) | 0.33 |
|  | | | | | | |
| AAPA-IgA- | 46 (21) | 175 (79) | **4.77 (3.39 - 6.74)** | **<0.0001** | 1 (ref) | **-** |
| AAPA-IgA+ | 20 (21) | 76 (79) | **4.77 (2.88 - 7.91)** | **<0.0001** | 1.00 (0.56 - 1.81) | 0.99 |
|  | | | | | | |
| AAPA-IgM- | 46 (21.3) | 170 (78.7) | **4.63 (3.28 - 6.55)** | **<0.0001** | 1 (ref) | **-** |
| AAPA-IgM+ | 20 (19.8) | 81 (80.2) | **5.08 (3.08 - 8.40)** | **<0.0001** | 1.10 (0.61 - 1.97) | 0.76 |

*odds ratio of univariate logistic regression. EAC: Leiden early arthritis clinic, AAPA: anti-acetylated protein antibodies, ACPA: anti-citrullinated protein antibodies, SE: shared epitope, OR: odds ratio, 95%CI: 95% confidence interval, na: not applicable

**Table S11.** Association of HLA SE, PTPN22 and HLA-DRB1 genes with AAPA-IgG in the EAC

|  | **All RA patients (n=361)** | | | | | **ACPA pos RA patients (n=182)** | | | | | | |
| --- | --- | --- | --- | --- | --- | --- | --- | --- | --- | --- | --- | --- |
| **PTPN22** | | | | | | | | | | | | |
|  | **PTPN22 neg n(%)** | **PTPN22 pos n(%)** | **OR (95% CI)** | **p-value** | **PTPN22 neg n(%)** | | | **PTPN22 pos n(%)** | **OR (95% CI)** | **p-value** | **OR (95% CI)** | **p-value** |
| Healthy controls | 338 (82.2) | 73 (17.76) | 1 (ref) | **-** | 338 (82.2) | | | 73 (17.76) | 1 (ref) | **-** |  |  |
| AAPA-IgG- | 183 (78.2) | 51 (21.8) | 1.29 (0.92 - 1.81) | 0.14 | 51 (75.0) | | | 17 (25.0) | 1.54 (0.87 - 2.75) | 0.14 | 1 (ref) | **-** |
| AAPA-IgG+ | 94 (74.0) | 33 (26.0) | 1.45 (0.91 - 2.32) | 0.11 | 83 (72.8) | | | 31 (27.2) | **1.67 (1.03 - 2.70)** | **0.04** | 1.12 (0.56 - 2.23) | 0.75 |
| **All RA patients (n=648)** | | | | | | | **ACPA pos RA patients** **(n=317)** | | | | | |
| **HLA-DRB1** | | | | | | | | | | | | |
|  | **HLA-DRB1*01 neg n(%)** | **HLA-DRB1*01 pos n(%)** | **OR (95% CI)** | **p-value** | **HLA-DRB1*01 neg n(%)** | | | **HLA-DRB1*01 pos n(%)** | **OR (95% CI)** | **p-value** | **OR (95% CI)** | **p-value** |
| Healthy controls | 948 (78) | 263 (22) | 1 (ref) | **-** | 948 (78) | | | 263 (22) | 1 (ref) | **-** |  |  |
| AAPA-IgG- | 313 (74) | 112 (26) | 1.29 (0.99 - 1.67) | 0.05 | 83 (73) | | | 30 (27) | 1.30 (0.84 - 2.02) | 0.24 |  |  |
| AAPA-IgG+ | 167 (75) | 56 (23) | 1.21 (0.87 - 1.68) | 0.26 | 150 (74) | | | 54 (26) | 1.29 (0.92 - 1.82) | 0.13 |  |  |
|  | | | | | | | | | | | | |
|  | **HLA-DRB1*03 neg n(%)** | **HLA-DRB1*03 pos n(%)** | **OR (95% CI)** | **p-value** | **HLA-DRB1*03 neg n(%)** | | | **HLA-DRB1*03 pos n(%)** | **OR (95% CI)** | **p-value** | **OR (95% CI)** | **p-value** |
| Healthy controls | 941 (78) | 270 (22) | 1 (ref) |  | 941 (78) | | | 270 (22) | 1 (ref) | **-** |  |  |
| AAPA-IgG- | 294 (69) | 131 (31) | **1.55 (1.21 - 1.99)** | **<0.0001** | 84 (74) | | | 29 (26) | 1.20 (0.77 - 1.87) | 0.41 | 1 (ref) | **-** |
| AAPA-IgG+ | 188 (84) | 35 (16) | **0.65 (0.44 - 0.95)** | **0.03** | 170 (83) | | | 34 (17) | 0.70 (0.47 - 1.03) | 0.07 | 0.58 (0.33 - 1.01) | 0.06 |
|  | | | | | | | | | | | | |
|  | **HLA-DRB1*04 neg n(%)** | **HLA-DRB1*04 pos n(%)** | **OR (95% CI)** | **p-value** | **HLA-DRB1*04 neg n(%)** | | | **HLA-DRB1*04 pos n(%)** | **OR (95% CI)** | **p-value** | **OR (95% CI)** | **p-value** |
| Healthy controls | 875 (72) | 336 (28) | 1 (ref) |  | 875 (72) | | | 336 (28) | 1 (ref) |  |  |  |
| AAPA-IgG- | 282 (66) | 143 (34) | **1.32 (1.04 - 1.67)** | **0.02** | 52 (46) | | | 61 (54) | 3.05 (2.07 - 4.52) | <0.0001 |  |  |
| AAPA-IgG+ | 102 (46) | 121 (54) | **3.09 (2.31 - 4.14)** | **<0.0001** | 94 (46) | | | 110 (54) | 3.05 (2.25 - 4.12) | <0.0001 |  |  |
|  | | | | | | | | | | | | |
|  | **HLA-DRB1*07 neg n(%)** | **HLA-DRB1*07 pos n(%)** | **OR (95% CI)** | **p-value** | **HLA-DRB1*07 neg n(%)** | | | **HLA-DRB1*07 pos n(%)** | **OR (95% CI)** | **p-value** | **OR (95% CI)** | **p-value** |
| Healthy controls | 973 (80) | 238 (20) | 1 (ref) |  | 973 (80) | | | 238 (20) | 1 (ref) | **-** |  |  |
| AAPA-IgG- | 358 (84) | 67 (16) | 0.77 (0.57 - 1.03) | 0.08 | **101 (89)** | | | **12 (11)** | **0.49 (0.26 - 0.90)** | **0.02** |  |  |
| AAPA-IgG+ | 191 (86) | 32 (14) | 0.68 (0.46 - 1.02) | 0.06 | 175 (86) | | | 29 (14) | 0.68 (0.45 - 1.03) | 0.07 |  |  |
|  | | | | | | | | | | | | |
|  | **HLA-DRB1*08 neg n(%)** | **HLA-DRB1*08 pos n(%)** | **OR (95% CI)** | **p-value** | **HLA-DRB1*08 neg n(%)** | | | **HLA-DRB1*08 pos n(%)** | **OR (95% CI)** | **p-value** | **OR (95% CI)** | **p-value** |
| Healthy controls | 1123 (93) | 88 (7) | 1 (ref) | - | 1123 (93) | | | 88 (7) | 1 (ref) | - |  |  |
| AAPA-IgG- | 412 (97) | 13 (3) | 0.40 (0.22 - 0.73) | 0.003 | 111 (98) | | | 2 (2) | **0.23 (0.06 - 0.95)** | **0.04** |  |  |
| AAPA-IgG+ | 216 (97) | 7 (3) | 0.41 (0.19 - 0.91) | 0.03 | 197 (97) | | | 7 (3) | **0.45 (0.21 - 0.99)** | **0.05** |  |  |
|  | | | | | | | | | | | | |
|  | **HLA-DRB1*09 neg n(%)** | **HLA-DRB1*09 pos n(%)** | **OR (95% CI)** | **p-value** | **HLA-DRB1*09 neg n(%)** | | | **HLA-DRB1*09 pos n(%)** | **OR (95% CI)** | **p-value** | **OR (95% CI)** | **p-value** |
| Healthy controls | 1183 (98) | 28 (2) | 1 (ref) |  | 1183 (98) | | | 28 (2) | 1 (ref) | **-** |  |  |
| AAPA-IgG- | 412 (97) | 13 (3) | 1.33 (0.68 - 2.60) | 0.40 | 109 (96) | | | 4 (4) | 1.55 (0.53 - 4.50) | 0.42 |  |  |
| AAPA-IgG+ | **212 (95)** | **11 (5)** | **2.20 (1.08 - 4.47)** | **0.03** | 195 (96) | | | 9 (4) | 1.95 (0.91 - 4.19 | 0.09 |  |  |
|  | | | | | | | | | | | | |
|  | **HLA-DRB1*10 neg n(%)** | **HLA-DRB1*10 pos n(%)** | **OR (95% CI)** | **p-value** | **HLA-DRB1*10 neg n(%)** | | | **HLA-DRB1*10 pos n(%)** | **OR (95% CI)** | **p-value** | **OR (95% CI)** | **p-value** |
| Healthy controls | 1180 (97) | 31 (3) | 1 (ref) |  | 1180 (97) | | | 31 (3) | 1 (ref) | **-** |  |  |
| AAPA-IgG- | 411 (97) | 14 (3) | 1.30 (0.68 - 2.46) | 0.43 | **104 (92)** | | | **9 (8)** | **3.29 (1.53 - 7.11)** | **0.002** | 1 (ref) | **-** |
| AAPA-IgG+ | **197 (88)** | **26 (12)** | **5.02 (2.92 - 8.64)** | **<0.0001** | **179 (88)** | | | **25 (12)** | **5.32 (3.07 - 9.21)** | **<0.0001** | 1.61 (0.73 - 3.59) | 0.24 |
|  | | | | | | | | | | | | |
|  | **HLA-DRB1*11 neg n(%)** | **HLA-DRB1*11 pos n(%)** | **OR (95% CI)** | **p-value** | **HLA-DRB1*11 neg n(%)** | | | **HLA-DRB1*11 pos n(%)** | **OR (95% CI)** | **p-value** | **OR (95% CI)** | **p-value** |
| Healthy controls | 999 (82) | 212 (18) | 1 (ref) |  | 999 (82) | | | 212 (18) | 1 (ref) | **-** |  |  |
| AAPA-IgG- | 353 (83) | 72 (17) | 0.96 (0.72 - 1.29) | 0.79 | 96 (85) | | | 17 (15) | 0.83 (0.49 - 1.43) | 0.51 | 1 (ref) | - |
| AAPA-IgG+ | 200 (90) | 23 (10) | 0.54 (0.34 - 0.86) | 0.008 | **185 (91)** | | | **19 (9)** | **0.48 (0.29 - 0.79)** | **0.004** | 0.58 (0.29 - 1.17) | 0.12 |
|  | | | | | | | | | | | | |
|  | **HLA-DRB1*12 neg n(%)** | **HLA-DRB1*12 pos n(%)** | **OR (95% CI)** | **p-value** | **HLA-DRB1*12 neg n(%)** | | | **HLA-DRB1*12 pos n(%)** | **OR (95% CI)** | **p-value** | **OR (95% CI)** | **p-value** |
| Healthy controls | 1154 (95) | 57 (5) | 1 (ref) |  | 1154 (95) | | | 57 (5) | 1 (ref) | **-** |  |  |
| AAPA-IgG- | 409 (96) | 16 (4) | 0.79 (0.45 - 1.39) | 0.40 | 112 (99) | | | 1 (1) | 0.18 0.03 - 1.32) | 0.09 |  |  |
| AAPA-IgG+ | 217 (97) | 6 (3) | 0.56 (0.24 - 1.31) | 0.18 | 198 (97) | | | 6 (3) | 0.61 (0.26 - 1.44) | 0.26 |  |  |
|  | | | | | | | | | | | | |
|  | **HLA-DRB1*13 neg n(%)** | **HLA-DRB1*13 pos n(%)** | **OR (95% CI)** | **p-value** | **HLA-DRB1*13 neg n(%)** | | | **HLA-DRB1*13 pos n(%)** | **OR (95% CI)** | **p-value** | **OR (95% CI)** | **p-value** |
| Healthy controls | 891 (74) | 3210 (26) | 1 (ref) | - | 891 (74) | | | 3210 (26) | 1 (ref) | - |  |  |
| AAPA-IgG- | **343 (81)** | **82 (19)** | **0.67 (0.51 - 0.87)** | **0.003** | **101 (89)** | | | **12 (11)** | **0.33 (0.18 - 0.61)** | **<0.0001** |  |  |
| AAP-IgG+ | **198 (89)** | **25 (11)** | **0.35 (0.23 - 0.54)** | **<0.0001** | **182 (89)** | | | **22 (11)** | **0.34 (0.21 - 0.53)** | **<0.0001** |  |  |
|  | | | | | | | | | | | | |
|  | **HLA-DRB1*14 neg n(%)** | **HLA-DRB1*14 pos n(%)** | **OR (95% CI)** | **p-value** | **HLA-DRB1*14 neg n(%)** | | | **HLA-DRB1*14 pos n(%)** | **OR (95% CI)** | **p-value** | **OR (95% CI)** | **p-value** |
| Healthy controls | 1128 (93) | 83 (7) | 1 (ref) | - | 1128 (93) | | | 83 (7) | 1 (ref) | - |  |  |
| AAPA-IgG- | 406 (96) | 19 (4) | 0.64 (0.38 - 1.06) | 0.08 | 110 (97) | | | 3 (3) | 0.37 (0.12 - 1.19) | 0.10 |  |  |
| AAPA-IgG+ | 213 (96) | 10 (4) | 0.64 (0.33 - 1.25) | 0.19 | 195 (96) | | | 9 (4) | 0.63 (0.31 - 1.27) | 0.19 |  |  |
|  | | | | | | | | | | | | |
|  | **HLA-DRB1*15 neg n(%)** | **HLA-DRB1*15 pos n(%)** | **OR (95% CI)** | **p-value** | **HLA-DRB1*15 neg n(%)** | | | **HLA-DRB1*15 pos n(%)** | **OR (95% CI)** | **p-value** | **OR (95% CI)** | **p-value** |
| Healthy controls | 882 (73) | 329 (27) | 1 (ref) |  | 882 (73) | | | 329 (27) | 1 (ref) | **-** |  |  |
| AAPA-IgG- | 319 (75) | 106 (25) | 0.89 (-.69 - 1.15) | 0.37 | 87 (77) | | | 26 (23) | 0.80 (0.51 - 1.26) | 0.67 |  |  |
| AAPA-IgG+ | 168 (75) | 55 (25) | 0.88 (0.63 - 1.22) | 0.44 | 155 (76) | | | 49 (24) | 0.85 (0.60 - 1.20) | 0.35 |  |  |
|  | | | | | | | | | | | | |
|  | **HLA-DRB1*16 neg n(%)** | **HLA-DRB1*16 pos n(%)** | **OR (95% CI)** | **p-value** | **HLA-DRB1*16 neg n(%)** | | | **HLA-DRB1*16 pos n(%)** | **OR (95% CI)** | **p-value** | **OR (95% CI)** | **p-value** |
| Healthy controls | 1184 (98) | 27 (2) | 1 (ref) |  | 1184 (98) | | | 27 (2) | 1 (ref) | **-** |  |  |
| AAPA-IgG- | 416 (98) | 9 (2) | 0.95 (0.44 - 2.03) | 0.89 | 111 (98) | | | 2 (2) | 0.79 (0.19 - 3.37) | 0.75 |  |  |
| AAPA-IgG+ | 220 (99) | 3 (1) | 0.60 (0.18 - 1.98) | 0.40 | 201 (99) | | | 3 (1) | 0.65 (0.20 - 2.18) | 0.49 |  |  |

When a difference in OR of >0.5 was found between AAPA-IgG- and AAPA-IgA+ patients was found or a significant association in the AAPA-IgG+ patients but not in the AAPA-IgG- patients, an additional logistic regression analyses was performed with the AAPA-IgG- as reference. All odds ratio are from univariate logistic regression.

EAC: Leiden early arthritis clinic, SERA: Scottish Early Rheumatoid Arthritis, AAPA: anti-acetylated protein antibodies, ACPA: anti-citrullinated protein antibodies, SE: shared epitope, PTPN22: Protein tyrosine phosphatase non-receptor type 22, HLA DRB1: HLA class II, histocompatibility antigen, DRB1 beta chain, OR: odds ratio, 95%CI: 95% confidence interval.

**Table S12.** Association of polygenic risk score (PRS) with AAPA-IgG in the EAC

|  | **PRS score mean (+/-SD)** | **n** | **OR (95% CI)*** | **p-value** | **OR (95% CI)*** | **p-value** |
| --- | --- | --- | --- | --- | --- | --- |
| Healthy controls | 1.02 (0.94) | 1015 | 1 (ref) | **-** |  |  |
| AAPA-IgG- | 1.18 (0.98) | 350 | **1.21 (1.09 - 1.35)** | **0.005** | 1 (ref) | **-** |
| AAPA-IgG+ | 1.23 (1.00) | 193 | **1.26 (1.07 - 1.47)** | **<0.0001** | 1.05 (0.88 - 1.26) | 0.56 |

*odds ratio of univariate logistic regression. EAC: Leiden early arthritis clinic, AAPA: anti-acetylated protein antibodies

**Table S13.** Interaction analysis of shared epitope and smoking with anti-acetylated protein antibodies and anti-citrullinated antibodies in the EAC & SERA

| **EAC** | | | | | | | | | |
| --- | --- | --- | --- | --- | --- | --- | --- | --- | --- |
|  | | **No SE alleles** | | | **Any SE alleles** | | **AP due to interaction (95%CI)** | **RERI due to interaction (95%CI)** | **S due to interaction (95%CI)** |
|  | | **Antibody +/ total (%)** | | **OR (95% CI)** | **Antibody +/ total (%)** | **OR (95% CI)** |  |  |  |
| **ACPA IgG** | | | | | | | |  |  |
| ACPA IgG - vs ACPA IgG +  Past/never smoking  Current smoking | | 48/171 (28)  18/51 (35) | | Referent  1.40 (0.72 - 2.72) | 178/293 (61)  73/102 (72) | 3.97 (2.64 - 5.96)  6.45 (3.74 - 11.12) | 0.32 (-0.32 - 0.96) | 2.09 (-2.53 - 6.70) | 1.62 (0.48 - 5.44) |
| **ACPA IgA & ACPA IgG** | | | | | | | |  |  |
| ACPA IgA - vs ACPA IgA +  Past/never smoking  Current smoking | | 25/171 (15)  9/51 (18) | | Referent  1.25 (0.54 - 2.98) | 92/293 (31)  52/102 (51) | 2.67 (1.64 - 4.37)  6.07 (3.42 - 10.79) | **0.51 (0.25 - 0.79)** | **3.15 (0.39 - 5.91)** | **2.64 (1.16 - 6.01)** |
| ACPA IgA-IgG- vs **ACPA** IgA-**IgG+**  Past/never smoking  Current smoking | | 23/146 (16)  9/42 (21) | | Referent  1.46 (0.62 - 3.45) | 87/201 (43)  21/50 (42) | 4.08 (2.41 - 6.90)  3.87 (1.89 - 7.93) | -0.17 (-0.95 - 0.61) | -0.67 (-3.45 - 2.11) | 0.81 (0.34 - 1.96) |
| ACPA IgA-IgG- vs **ACPA IgA+IgG+**  Past/never smoking  Current smoking | | 25/148 (17)  9/42 (21) | | Referent  1.34 (0.57 - 3.15) | 91/205 (44)  52/81 (64) | 3.93 (2.36 - 6.54)  8.82 (4.72 - 16.49) | **0.52 (0.23 - 0.81)** | 4.55 (-0.09 - 9.20) | **2.39 (1.15 - 4.99)** |
| **ACPA** IgA-**IgG+** vs **ACPA IgA+IgG+**  Past/never smoking  Current smoking | | 25/48 (52)  9/18 (50) | | Referent  0.92 (0.31 - 2.72) | 91/178 (51)  52/73 (71) | 0.96 (0.51 - 1.82)  2.28 (1.07 - 4.87) | **0.61 (0.11 - 1.12)** | 1.40 (-0.08 - 2.87) | 10.86 (0-3391021) |
| **AAPA IgG** | |  | |  |  |  |  |  |  |
| AAPA IgG - vs AAPA IgG +  Past/never smoking  Current smoking | | 31/171 (18)  11/51 (22) | | Referent  1.24 (0.57 - 2.68) | 116/293 (40)  49/102 (48) | 2.96 (1.88 - 4.66)  4.18 (2.41 - 7.24) | 0.23 (-0.17 - 0.64) | 0.97 (-0.98 - 2.92) | 1.44 (0.69 - 3.04) |
| **AAPA IgA & AAPA IgG** | | | | | | | |  |  |
| AAPA IgA - vs AAPA IgA +  Past/never smoking  Current smoking | | 16/171 (9)  12/51 (24) | | Referent  2.98 (1.30 - 6.81) | 50/293 (17)  32/102 (31) | 1.99 (1.10 - 3.62)  4.43 (2.57 - 7.63) | 0.10 (-0.70 - 0.90) | 0.45 (-3.02 - 3.93) | 1.15 (0.35 - 3.84) |
| AAPA IgA-IgG- vs **AAPA** IgA-**IgG+**  Past/never smoking  Current smoking | | 21/155 (14)  4/39 (10) | | Referent  0.73 (0.24 - 2.26) | 78/243 (32)  18/70 (26) | 3.02 (1.77 - 5.14)  2.21 (1.09 - 4.48) | -0.24 (-1.10 - 0.62) | -0.54 (-2.31 - 1.23) | 0.69 (0.21 - 2.25) |
| AAPA IgA-IgG+ vs **AAPA IgA+IgG+**  Past/never smoking  Current smoking | | 10/144 (7)  7/42 (17) | | Referent  2.68 (0.95 - 7.55) | 38/203 (19)  31/83 (37) | 3.09 (1.48 - 6.42)  7.99 (3.66 - 17.45) | 0.40 (-0.09 - 0.90) | 3.22 (-2.27 - 8.71) | 1.86 (0.67 - 5.13) |
| ACPA pos RA  AAPA IgA-AAPA IgG- vs AAPA IgA+AAPA IgG+  Past/never smoking  Current smoking | | 10/47 (37)  7/12 (58) | | Referent  2.38 (0.99 - 5.75) | 38/96 (40)  31/53 (57) | 1.11 (0.28 - 4.46)  2.29 (0.89 - 5.92) | -0.09 (-1.65 - 1.47) | -0.20 (-3.7 - 3.33) | 0.86 (0.08 - 9.77) |
|  | | | | | | | | | |
| **SERA** | | | | | | | | | |
|  | **No SE alleles** | | | | **Any SE alleles** | | **AP due to interaction (95%CI)** | **RERI due to interaction (95%CI)** | **S due to interaction (95%CI)** |
|  | **Antibody +/ total (%)** | | **OR (95% CI)** | | **Antibody +/ total (%)** | **OR (95% CI)** |  |  |  |
| **ACPA IgG** | | | | | | | |  |  |
| ACPA IgG - vs ACPA IgG +  Past/never smoking  Current smoking | 47/83 (57)  14/17 (82) | | Referent  3.57 (0.95 - 13.39) | | 146/173 (84)  61/65 (94) | 4.14 (2.28 - 7.53)  11.68 (3.89 - 35.12) | 0.43 (-0.29 - 17.82) | 4.96 (-7.89 - 17.82) | 1.87 (0.47 - 7.50) |
|  | **No SE alleles** | | | | **Any SE alleles** | | **AP due to interaction (95%CI)** | **RERI due to interaction (95%CI)** | **S due to interaction (95%CI)** |
|  | **Antibody +/ total (%)** | | **OR (95% CI)** | | **Antibody +/ total (%)** | **OR (95% CI)** |  |  |  |
| **ACPA IgA & ACPA IgG** | | | | | | | |  |  |
| ACPA IgA - vs ACPA IgA +  Past/never smoking  Current smoking | 11/83 (13)  5/17 (29) | | Referent  2.73 (0.8 - 9.25) | | 46/173 (27)  30/65 (46) | 2.37 (1.16 - 4.86)  5.61 (2.52 - 12.49) | 0.27 (-0.38 - 0.92) | 1.51 (-2.53 - 5.55) | 1.49 (0.47 - 4.73) |
| ACPA IgA-IgG- vs ACPA IgA-IgG+  Past/never smoking  Current smoking | 37/72 (51)  9/12 (75) | | Referent  2.84 (0.71 - 11.35) | | 100/127 (79)  31/35 (89) | 3.5 (1.87 - 6.56)  7.33 (2.35 - 22.9) | 0.27 (-0.66 - 1.21) | 1.99 (-6.64 - 10.62) | 1.46 (0.32 - 6.69) |
| ACPA IgA-IgG- vs ACPA IgA+IgG+  Past/never smoking  Current smoking | 10/45 (22)  5/8 (63) | | Referent  5.83 (1.18 - 28.74) | | 46/73 (63)  30/34 (88) | 5.96 (2.55 - 13.93)  26.25 (7.46 - 92.35) | **0.59 (0.04 - 1.13)** | 15.45 (-15.06 - 45.96) | 2.58 (0.63 - 10.6) |
| ACPA IgA-IgG+ vs ACPA IgA+IgG+  Past/never smoking  Current smoking | 10/47 (21)  9/14 (64) | | Referent  2.06 (0.56 - 7.52) | | 46/146 (32)  30/61 (49) | 1.7 (0.78 - 3.72)  3.58 (1.51 - 8.46) | 0.23 (-0.55 - 1.01) | 0.82 (-2.12 - 3.76) | 1.47 (0.3 - 7.16) |
| **AAPA IgG** | | | | | | | | | |
| AAPA IgG - vs AAPA IgG +  Past/never smoking  Current smoking | 14/83 (17)  6/17 (35) | | Referent  2.69 (0.85 - 8.48) | | 71/173 (41)  27/65 (42) | 3.43 (1.79 - 6.57)  3.5 (1.64 - 7.47) | -0.46 (-1.57 - 0.64) | -1.62 (-5.29 - 2.05) | 0.61 (0.23 - 1.62) |
| **AAPA IgA & AAPA IgG** | | | | | | | |  |  |
| AAPA IgA - vs AAPA IgA +  Past/never smoking  Current smoking | 2/83 (4)  2/17 (12) | | Referent  5.4 (0.71 - 41.36) | | 8/173 (5)  11/65 (17) | 1.96 (0.41 - 9.46)  8.25 (1.76 - 38.69) | 0.23 (-0.88 - 1.34) | 1.89 (-7.89 - 11.66) | 1.35 (0.24 - 7.48) |
| AAPA IgA-IgG- vs AAPA IgA-IgG+  Past/never smoking  Current smoking | 13/81 (16)  4/15 (27) | | Referent  1.9 (0.52 - 6.9) | | 63/165 (38)  16/54 (30) | 3.23 (1.65 - 6.32)  2.2 (0.96 - 5.06) | -0.88 (-2.45 - 0.69) | -1.93 (-5.16 - 1.3) | 0.38 (0.1 - 1.44) |
| AAPA IgA-IgG+ vs AAPA IgA+IgG+  Past/never smoking  Current smoking | 1/14 (7)  2/6 (33) | | Referent  6.5 (0.46 - 91.92 | | 8/71 (11)  11/27 (41) | 1.65 (0.19 - 14.36)  8.94 (1.02 - 78.59) | 0.2 (-1.2 - 1.6) | 1.79 (-11.36 - 14.93) | 1.29 (0.17 - 9.94) |
| ACPA pos RA  AAPA-AAPA- vs AAPA+AAPA+  Past/never smoking  Current smoking | 1/69 (1)  2/13 (2) | | Referent  12.36 (1.03 - 148.05) | | 8/110 (7)  11/49 (2) | 5.33 (0.65 - 43.57)  19.68 (2.45 - 158.25) | 0.15 (-0.97 - 1.28) | 2.99 (-20.5 - 26.47) | 1.19 (0.29 - 4.88) |

All odds ratio are from univariate logistic regression. EAC: Leiden early arthritis clinic, SERA: AAPA: anti-acetylated protein antibodies, ACPA: anti-citrullinated protein antibodies, SE: shared epitope, OR: odds ratio, 95%CI: 95% confidence interval. AP: attributable proportion due to interaction, RERI: Relative excess risk due to interaction, S: Synergy index (S), RA: rheumatoid arthritis

**Supplementary figures**

**Figure S1.** flowchart systematic literature review smoking and ACPA isotypes

**
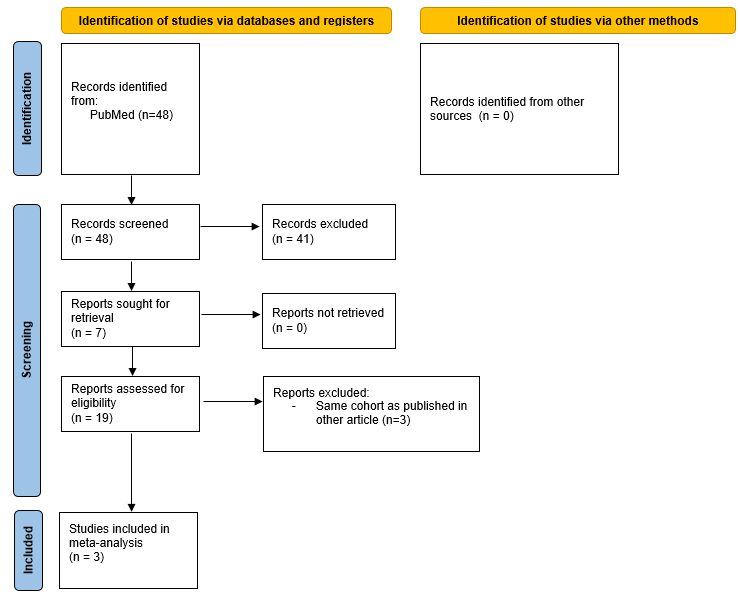
**

**Figure S2.** flowchart systematic literature review shared epitope and AAPA-IgG


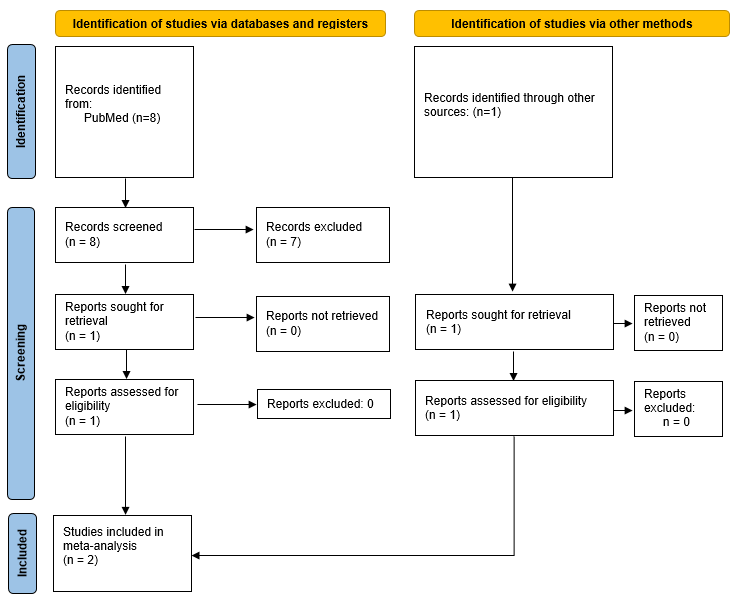


**Figure S3.** Association of smoking corrected for autoantibody levels in the EAC. A. Association of smoking with ACPA-IgA and ACPA-IgG corrected for respectively ACPA-IgG positivity and ACPA-IgA positivity*. B. Association of smoking with AAPA-IgA and AAPA-IgG corrected for respectively AAPA-IgG levels and AAPA-IgA levels. C. Association of smoking with CarP-IgA and CarP-IgG corrected for respectively CarP-IgG levels and CarP-IgA levels. D. Association of smoking with RF-IgA and RF-IgM corrected for respectively RF IgM levels and RF IgA levels.
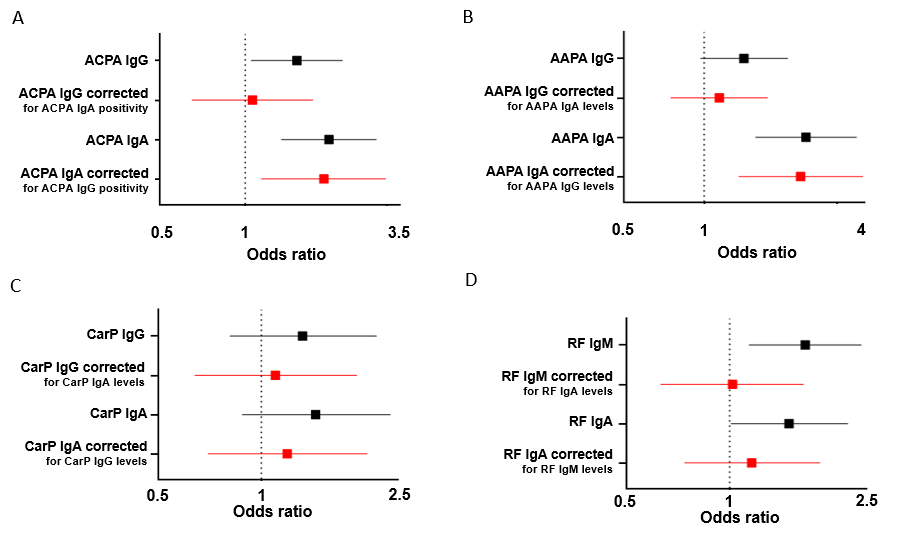


ACPA: anti-citrullinated protein antibodies, AAPA: anti-acetylated protein antibodies, CarP: anti-carbamylated protein antibodies, RF: rheumatoid factor**,** EAC: Leiden early arthritis clinic

* Because of the high collinearity between ACPA-IgA and ACPA-IgG levels, correction for levels was not possible for ACPA-isotypes and instead we corrected for isotype-positivity

**Figure S4.** Sub meta-analysis combining data from the EAC, SERA and tREACH of smoking with ACPA-IgG and ACPA-IgA. Smoking was defined as current versus never and past. A. Meta-analysis for the association of smoking: ACPA-IgG- ACPA-IgA- versus ACPA-IgG+ ACPA-IgA- B. Association of smoking: ACPA-IgG- ACPA-IgA- versus ACPA-IgG- ACPA-IgA+ (no meta-analysis possible since this was examined in one study) C. Meta-analysis for the association of smoking: ACPA-IgG- ACPA-IgA- versus ACPA-IgG+ ACPA-IgA+ D. Meta-analysis for the association of smoking: ACPA-IgG+ ACPA-IgA- versus ACPA-IgG+ ACPA-IgA+.

**
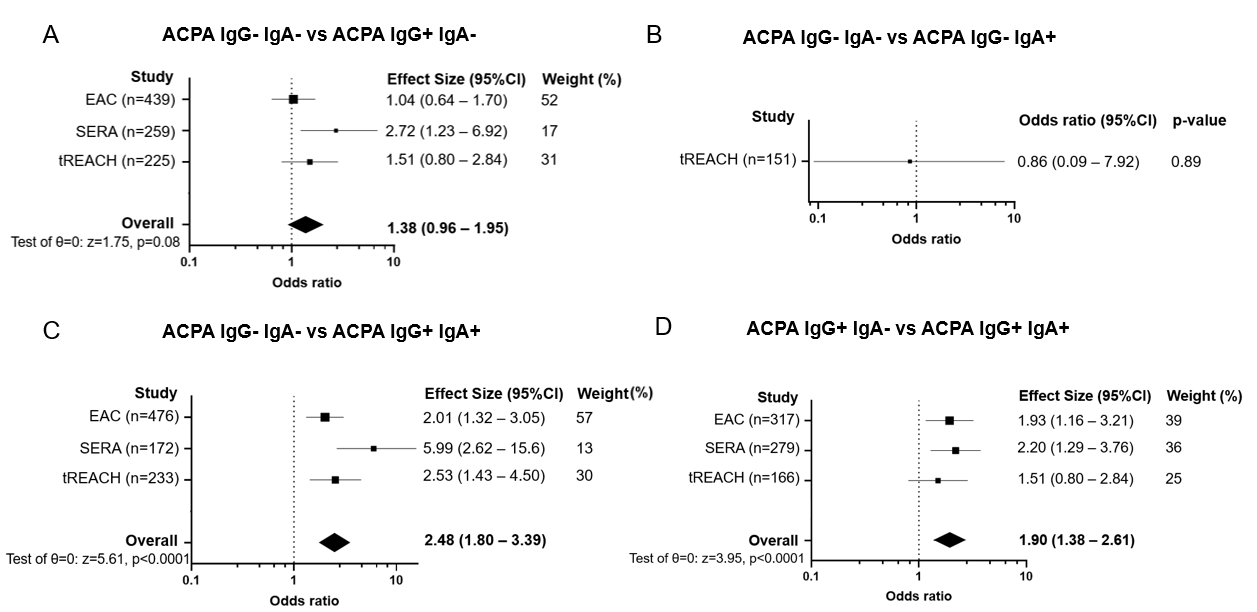
**

ACPA: anti-citrullinated protein antibodies. EAC: Leiden early arthritis clinic, EIRA: Epidemiological Investigations of RA^9^, SERA: Scottish Early Rheumatoid Arthritis, TIRA 1: Early Intervention in RA 1^8^, TIRA 2: Early Intervention in RA 2^9^, tREACH: treatment in the Rotterdam Early Arthritis Cohort

**Figure S5.** Sub meta-analysis combining data from the EAC, SERA and tREACH with the SLR of smoking ever with ACPA-IgG and ACPA-IgA. Smoking was defined as ever versus never. A. Meta-analysis for the association of smoking: ACPA-IgG- ACPA-IgA- versus ACPA-IgG+ ACPA-IgA- B. Association of smoking: ACPA-IgG- ACPA-IgA- versus ACPA-IgG- ACPA-IgA+ (no meta-analysis possible since this was examined in one study) C. Meta-analysis for the association of smoking: ACPA-IgG- ACPA-IgA- versus ACPA-IgG+ ACPA-IgA+ D. Meta-analysis for the association of smoking: ACPA-IgG+ ACPA-IgA- versus ACPA-IgG+ ACPA-IgA+.

**
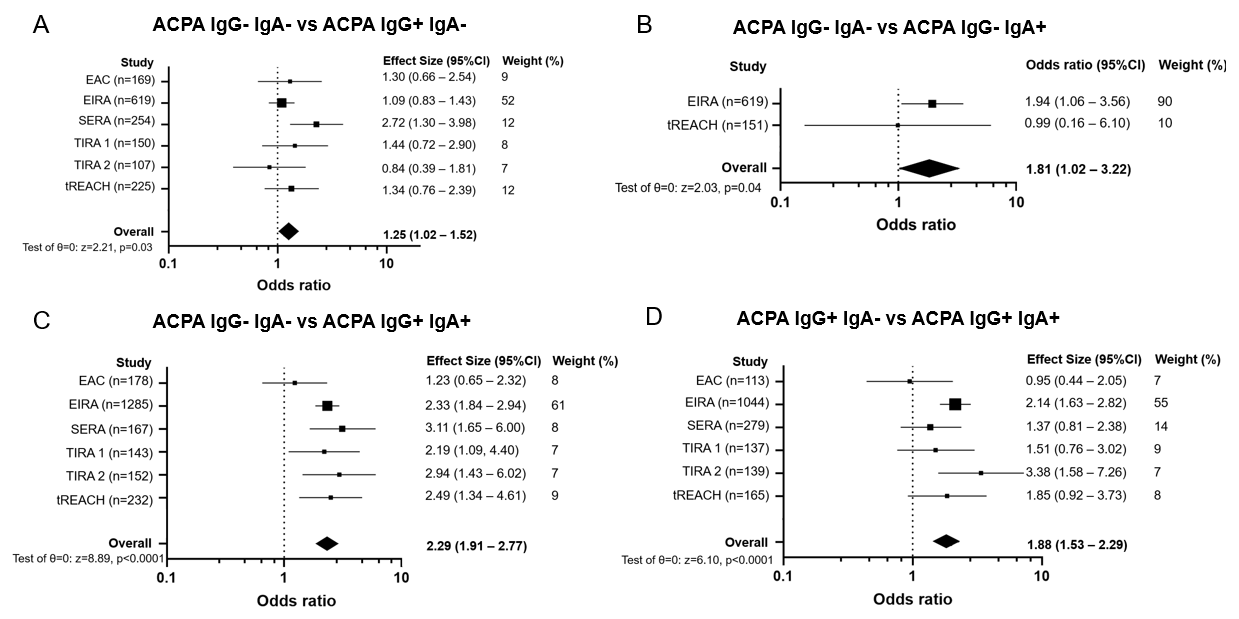
**

ACPA: anti-citrullinated protein antibodies. EAC: Leiden early arthritis clinic, EIRA: Epidemiological Investigations of RA^9^, SERA: Scottish Early Rheumatoid Arthritis, TIRA 1: Early Intervention in RA 1^8^, TIRA 2: Early Intervention in RA 2^9^, tREACH: treatment in the Rotterdam Early Arthritis Cohort

**Figure S6.** Interaction of SE and smoking with ACPA in the EAC. In the top bars the single effects (odds ratio’s) of smoking and SE are depicted, while in the bottom bars the effect of their combined presence is shown. When the effect of the simultaneous presence of these risk factors leads to a substantially larger odds ratio than the sum of their single effects, this indicates the presence of interaction. A. Interaction of smoking and SE in ACPA IgG+IgA- versus ACPA IgG-IgA- patients. B. Interaction of smoking and SE in ACPA IgG+IgA+ versus ACPA IgG-IgA- patients. C . Interaction of smoking and SE in AAPA IgG+IgA- versus AAPA IgG-IgA- patients. D. Interaction of smoking and SE in AAPA IgG+IgA+ versus ACPA IgG-IgA- patients E. . Interaction of smoking and AAPA IgG+IgA+ versus AAPA IgG-IgA- SE in ACPA IgG positive patients.


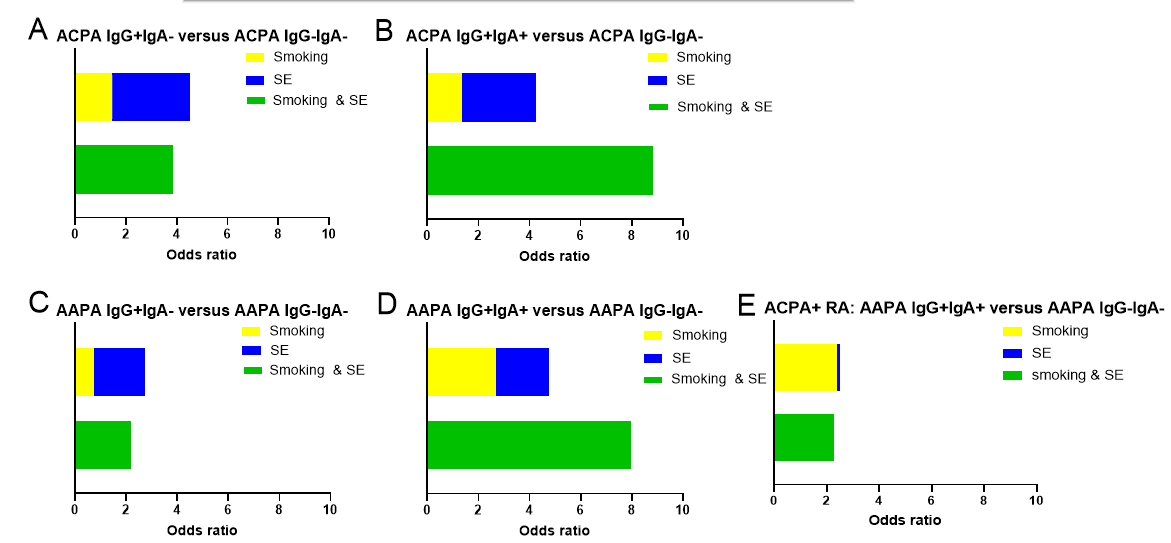
 EAC: Leiden early arthritis clinic, AAPA: anti-acetylated protein antibodies, ACPA: anti-citrullinated protein antibodies, EAC: Leiden early arthritis clinic

**References**

1. van Aken, J., et al., *The Leiden Early Arthritis Clinic.* Clin Exp Rheumatol, 2003. **21**(5 Suppl 31): p. S100-5.

2. de Jong, P.H., et al., *Induction therapy with a combination of DMARDs is better than methotrexate monotherapy: first results of the tREACH trial.* Ann Rheum Dis, 2013. **72**(1): p. 72-8.

3. Claessen, S.J., et al., *Use of risk stratification to target therapies in patients with recent onset arthritis; design of a prospective randomized multicenter controlled trial.* BMC Musculoskelet Disord, 2009. **10**: p. 71.

4. Dale, J., et al., *The Scottish Early Rheumatoid Arthritis (SERA) Study: an inception cohort and biobank.* BMC Musculoskelet Disord, 2016. **17**(1): p. 461.

5. Smith, B.H., et al., *Generation Scotland: the Scottish Family Health Study; a new resource for researching genes and heritability.* BMC Med Genet, 2006. **7**: p. 74.

6. Verpoort, K.N., et al., *Isotype distribution of anti-cyclic citrullinated peptide antibodies in undifferentiated arthritis and rheumatoid arthritis reflects an ongoing immune response.* Arthritis Rheum, 2006. **54**(12): p. 3799-808.

7. Nijjar, J.S., et al., *The impact of autoantibodies against citrullinated, carbamylated, and acetylated peptides on radiographic progression in patients with new-onset rheumatoid arthritis: an observational cohort study.* Lancet Rheumatol, 2021. **3**(4): p. e284-e293.

8. Svärd, A., et al., *A comparison between IgG- and IgA-class antibodies to cyclic citrullinated peptides and to modified citrullinated vimentin in early rheumatoid arthritis and very early arthritis.* J Rheumatol, 2011. **38**(7): p. 1265-72.

9. Svärd, A., et al., *Associations with smoking and shared epitope differ between IgA- and IgG-class antibodies to cyclic citrullinated peptides in early rheumatoid arthritis.* Arthritis Rheumatol, 2015. **67**(8): p. 2032-7.

10. de Moel, E.C., et al., *Geo-epidemiology of autoantibodies in rheumatoid arthritis: comparison between four ethnically diverse populations.* Arthritis Res Ther, 2023. **25**(1): p. 37.

11. Grönwall, C., et al., *A Comprehensive Evaluation of the Relationship Between Different IgG and IgA Anti-Modified Protein Autoantibodies in Rheumatoid Arthritis.* Front Immunol, 2021. **12**: p. 627986.
